# Supplementary material for: Mental Health Symptom Reduction Using Digital Therapeutics Care Informed by Genomic SNPs and Gut Microbiome Signatures
Source: J Pers Med. 2022 Jul 28;12(8):1237. doi: 10.3390/jpm12081237 (PMC9409755; doi:10.3390/jpm12081237)
Supplement: Supplementary file 1 [file jpm-12-01237-s001.zip › Mental Health Symptom Reduction Using Digital Therapeutics Care -- JPM MDPI -- Supp Information - 2022-07-27.pdf]

Supplementary Information

Mental Health Symptom Reduction Using Digital Therapeutics Care Informed by Genomic SNPs and Gut Microbiome Signatures

Inti Pedroso[1ⱡ], Shreyas V. Kumbhare[1ⱡ], Bharat Joshi[1], Santosh K. Saravanan[1], Dattatray S. Mongod[2], Simitha Singh-Rambiritch[1], Tejaswini Uday[1], Karthik M. Muthukumar[1], Carmel Irudayanathan[1], Chandana Reddy-Sinha[1], Parambir S. Dulai[3], Ranjan Sinha[1*], Daniel E. Almonacid[1*]

[1] Digbi Health, Mountain View, California, USA
[2] National Centre for Cell Science, Pune, Maharashtra, India
[3] Division of Gastroenterology, Northwestern University, Chicago, Illinois, USA

ⱡ Authors contributed equally.
* Corresponding authors. E-mail addresses: almonacid@digbihealth.com, ranjan@digbihealth.com

Keywords: Anxiety; Depression; Insomnia; Gut-brain-axis; Multi-omic models; Non-pharmacological treatment

Supplementary Methods **4**
Study rationale 4
Participant enrollment, intervention, and phenotype data collection 4
Sample collection and processing: Genome SNP array and gut microbiome sequencing 5
Microbiome data analyses 5
Genetic data analyses 6
Statistical analysis 6

Supplementary Table Legends **8**
Table S1. Demographic data of participants and survey information used in the study. 8
Table S2. Summary characteristics of the cohort. 8
Table S3. Genetic scores used in the study. 8
Table S4. Summary characteristics of the cohorts studied for the improvement of anxiety, depression, and insomnia. 8
Table S5. Summary statistics of genetic and microbiome factors significantly associated with the study outcomes. 8
Table S6. Statistical associations with improvement in anxiety. 8
Table S7. Statistical associations with improvement in depression. 8
Table S8. Statistical associations with improvement in insomnia. 8
Table S9. Summary characteristics of the cohorts studied at baseline. 8
Table S10. Statistical associations with anxiety or depression at baseline. 9
Table S11. Statistical associations with sleep problems at baseline. 9
Table S12. Model fit of combined models of demographics, microbiome, and genetic predictors. 9
Table S13. Effect of medications on the gut microbiome. 9

Supplementary Figures **11**
Figure S1. Study design and cohort sample size for each outcome. 11
Figure S2. Changes in symptom intensity after the intervention. 12
Figure S3. Boxplot of microbiome features associated with improvement on anxiety. 13
Figure S4. Boxplot of genetic scores associated with improvement on anxiety. + 14
Figure S5. Boxplot of microbiome features associated with improvement on depression. 15
Figure S6. Boxplot of genetic scores associated with improvement on depression. 16
Figure S7. Boxplot of microbiome features associated with improvement on insomnia. 17
Figure S8. Boxplot of genetic scores associated with improvement on insomnia. 18
Figure S9. Community-level comparison of the gut microbiome of 328 individuals with and without reported anxiety or depression at baseline. 19
Figure S10. Boxplot of genetic scores associated with anxiety or depression at baseline. 20
Figure S11. Boxplot of microbiome features associated with sleep problems at baseline. 21
Figure S12 Comparison of models for all outcomes of the study based on demographic, microbiome and genetic factors. 22

Figure S13. Comparison of functional pathway associations with outcomes at baseline versus improvement at follow-up. 23
Figure S14. Comparison of bacterial genera associations with outcomes at baseline versus improvement at follow-up. 25
Figure S15. Comparison of genetic scores associations with outcomes at baseline versus improvement at follow-up. 27
References 29

Supplementary Methods

Study rationale

This study aimed to identify genetic and gut microbiome factors associated with mental health improvement after a dietary and lifestyle intervention. We included the study subjects that reported mental health conditions, particularly anxiety or depression when initiating the intervention. Study subjects were asked to rate their improvement in mental health after they had achieved at least 2% body weight loss through the digital therapeutic program. This low body weight loss threshold allowed us to have a broad range of body weight loss in this cohort. Additionally, our previously published research indicated that two thirds of high BMI individuals lost at least 2% body weight 120 days after initiating the intervention. As genetic information, we considered genetic scores for traits that are known comorbidities of anxiety, depression, and obesity (see details below). We added to the outcomes of the study sleep problems at baseline and insomnia at follow-up. Both are integral parts of anxiety and depression and are known to be associated with digestive and gut issues, like functional gastrointestinal disorders. As for the gut microbiome, we considered taxonomic annotation summarized at the level of genera and predicted functional pathways previously linked with mental health (see details below).

Participant enrollment, intervention, and phenotype data collection

Study participants were recruited from February 2020 to October 2021 among those who achieved 2% or more body weight loss from the date when enrolled in the Digbi Health personalized digital care program. Participants filled an online questionnaire regarding anxiety or depression, and problems at baseline. Those who indicated a positive answer to having anxiety or depression were asked to rate the intensity of their anxiety and depression on a scale from 0 (minimum) to 5 (maximum). Those that indicated a positive answer about having sleep problems at baseline were asked to rate the intensity of their insomnia on a scale from 0 (minimum) to 5 (maximum). At baseline participants provided information on the presence or absence of symptoms associated with FGIDs, prescribed and over-the-counter medications or supplements, alcohol intake, recreational drug usage, and demographic information, including age, gender, and height. Body mass index at baseline and follow-up was calculated using the closest weight measurement to the enrollment date and date on which the survey was answered (within ± 14 days). Individuals were classified as having an FGID if they reported one or more of the following seven conditions: constipation, diarrhea, gassiness, bloating, heartburn/acid reflux, abdominal pain (cramping/belly pain) and IBS [1, 2].

Participants consuming either antidepressants, anti-anxiolytics, antibiotics, or antimycotics, were identified by mapping the self-reported medications names to the chembl database using the database API [3] and dentified medications prescribed for depression (N06AA, N06AB, and N06AX), anxiety (N05B) and antibiotics and antimycotics (J01B, J01C, J01D, J01E, J01F, J01G, J01M, J01R, J01X, J02, and J04) using ATC codes (https://www.whocc.no/atc_ddd_index/).

Digbi Health's intervention has been described elsewhere [4]. In a nutshell, personalization of plans is achieved by analyzing participants' genetics, gut bacteria, lifestyle, and demographics. Based on these data, the program encourages participants to make incremental lifestyle changes focused on reducing sugar consumption and timing meals to optimize insulin sensitivity, reduce systemic inflammation by identifying possibly inflammatory and anti-inflammatory nutrients, and increase fiber diversity to improve gut health. Behavioral changes are implemented with the help of virtual health coaching and the app, ensuring they are habit-forming.

Sample collection and processing: Genome SNP array and gut microbiome sequencing

Subjects self-collected saliva samples using buccal swabs (Mawi Technologies iSwab DNA collection kit, Model no. ISWAB-DNA-1200) and fecal samples using fecal swabs (Mawi Technologies iSWAB Microbiome collection kit, Model no. ISWAB-MBF-1200). Sample collection was completed by following standardized directions provided to all subjects in an instruction manual. Saliva DNA extraction, purification, and genotyping using Affymetrix's Direct to Consumer Array version 2.0 ("DTC") on the Affymetrix GeneTitan platform was performed by Akesogen Laboratories in Atlanta, GA. Genotype calling was performed using Axiom Analysis Suite Software version 5.1.1. Genotypes were set to 'missing' if the sample did not meet the confidence threshold for making the call or if the probeset did not fall into a recommended category. Specific details about the probeset metrics can be found in the Axiom Analysis Suite User Guide or the Genotyping Data Analysis Guide (ThermoFisher Scientific 2022). Sample processing of baseline (pre-intervention) fecal samples was followed by the 16S rRNA gene amplicon sequencing performed at Akesogen Laboratories in Atlanta, GA. DNA extraction was performed using Qiagen MagAttract Power Microbiome DNA Kit on an automated liquid handling DNA extraction instrument.

Microbiome data analyses

The V3-V4 region of the 16S rRNA gene was amplified and sequenced on the Illumina MiSeq platform using 2 x 300 bp paired-end sequencing [5]. Sequence reads were demultiplexed, and ASVs generated using DADA2 in QIIME2 version 2020.8 [6]. Primers and low-quality bases (Q <30) were trimmed off the reads from forward and reverse reads. Taxonomic assignment was performed using the vsearch consensus method against the 99% non-redundant Silva 138 reference database [7]. We excluded hits to Mitochondria, Chloroplast, Eukaryota, and unassigned taxa at the phylum level [8]. Six samples for participants who reported antibiotic consumption were excluded from the downstream analysis. Any ASV not seen more than once in at least 10% of the samples were filtered out to remove ASVs with a small mean and trivially large coefficient of variation [8]. The abundance matrix was rarefied at even depth (n=36,000 reads per sample with 500 iterations) using the 'q2-repeat-rarefy' plugin from QIIME2 [9]. The abundance matrix was then agglomerated at the genus level, resulting in 178 taxa across 344 samples. We further filtered any taxa with read counts less than 10. Furthermore, the raw abundance data were subjected to centered-log ratios (CLR) transformation [10].

The microbial functional prediction was performed using the q2-picrust2 plugin (version 2021.2) in QIIME2 [11]. The abundance matrix comprising KEGG Orthologs was then used to obtain the abundance of specific pathways related to neuroactive metabolites produced by gut microbes, described as 'Gut-Brain Modules' (GBMs) in an earlier study [12]. Predicted gene abundance encoding these metabolites was derived using the Omixer-RPM package (version 0.3.2) [13].

Genetic data analyses

Probe level genotype calls were unified at the variant level and formatted in VCF format, and multiallelic sites with discordant genotype calls among different probes were set as missing. VCF files were left normalized with bcftools version 1.14 (using htslib version 1.14). Beagle version 5.3 [14, 15] was used for phasing and imputation using the 1KG project as reference [16]. Processing of the 1KG data for imputation included only SNPs and Indels, removing sites with allele counts <2 and left normalization with bcftools, and converting filtered and phased VCF files to bref3 format using the bref3.28Jun21.220.jar script provided by the Beagle software suite. We included 13,478,023 variants on downstream analyses deriving from sites imputed with r^2>=0.8 or chip-genotyped. All analyses were orchestrated by a SnakeMake pipeline [17].

We merged our un-imputed genotype data with the 1KG data, including only sites with MAF > 1% and genotype missingness < 10%. We performed PCA analysis and calculated the first 20 PCs using Plink2 [18] on the combined dataset. We focused on the main five super populations defined by the 1KG project to estimate the sample's ancestry composition. We trained a random forest classifier as implemented on scikit-learn [19] with max depth = 8 and number of trees = 100 using 75% and 25% of the dataset for training and test, respectively. We obtained a Matthew's correlation coefficient of 0.98 showing good performance as has been reported elsewhere for this strategy [20].

Twenty-three traits were selected based on being digestive system traits comorbid with mental health disorders, anxiety and sleep in particular, or mental health disorders that are known comorbidities of digestive system conditions focused on IBS, IBD, and obesity [21, 22]. We reviewed each trait's genetic and genome-wide association studies and extracted summary statistics, including chromosome, position, effect allele, effect size, and ancestry of discovery and replication populations. Table S2 provides additional information for each trait and associated references as data sources. Genetic scores were calculated by multiplying the beta value or logarithm of the odds ratio by the number of risk alleles each individual had and the mean overall genetic variants included on the panel. All genetic scores were coded to be interpreted such that a larger genetic score is associated with increasing inherited genetic predisposition to the condition.

Statistical analysis

We used logistic regression for sleep problems at baseline and anxiety or depression at baseline. We used Poisson regression to improve intensity scores, with the outcome being the intensity at T1 and offset being the intensity at T0. In all models, we included as demographic variables FGID: binary, the self-reported status of the previous diagnosis of a functional

gastrointestinal disorder; gender: binary, male or female; BMI at T0: continuous variable; Age: continuous variable; weight loss: categorical variable, categorized as those with no change, lost 0 to 5%, 5-10% or more than 10% of their body weight at T1 in relation to T0; and five principal components (continuous variable) calculated using the genetic ancestry analyses described previously. Linear regression was fitted using the statsmodels python package [23] v0.13.2 using the Binomial and Poisson families with the HC3 covariance matrix as recommended elsewhere [24]. These regression models identified microbiome and genetic factors measured at baseline that are associated with increased prevalence of mental health at baseline and different levels of improvement in mental health at follow-up. On the logistic models a regression coefficient greater than zero is interpreted as an increasing prevalence of self-reported illness with an increasing abundance of microbiome factors or a higher value of the genetic scores. On the Poisson regression models, a regression coefficient greater than zero is interpreted as a higher abundance of microbiome factors or a higher value of genetic scores being associated with less than average improvement in the outcome.

We applied correction for multiple hypothesis tests using the False Discovery Rate (FDR) [25], as implemented on thestatsmodels python package, and selected statistically significant results with an FDR ≤ 0.15.

We performed model comparisons between four different models by building regression models for demographic predictors only (D), demographic and microbiome predictors (D+M), demographic and genetic predictors (D+M), and all three sets of predictors (D+M+G). For microbiome and genetic predictors, we included in the models' variables identified on univariate analyses with an FDR ≤ 0.15. We performed a singular value decomposition (SVD) analysis with the microbiome variables, bacterial genera, and functional pathways to avoid collinearity in the regression models. We used the singular vectors as predictors on the regression models. We selected the same number of singular vectors as the number of microbiome variables used for the SVD. We report adjusted Cox-Snell pseudo r-squared values, which allow comparison between models with a different number of predictors; in particular, we used Pratt's method [26], which is recommended for cases where the ratio between the number of samples and predictors is less than 100 [27]. To estimate the mean, median, standard deviation, and percentiles of the pseudo r-squared values, we performed bootstrap with 1001 bootstrap replicates. The bootstrap analysis is not meant for hypothesis testing but to provide uncertainty estimated around the pseudo r-squared reported.

We tested the effect of potential confounders: medications and alcohol consumption, on the association of gut microbiome with mental health outcomes, by performing multivariate analysis using PERMANOVA with 999 bootstrap iterations based on Bray-Curtis dissimilarity with the vegan package in R [28]. Age explained the highest variation in the microbiome at baseline, and hence to reduce its confounding effect, PERMANOVA models were run by controlling for age by stratifying it as blocks (strata=age). We additionally performed linear regression models with CLR transformed taxa abundances as outcomes and included potential confounders and their interaction with mental health as predictors.

Example code for the regression statistical analyses

The following blocks of code and data is provided with the porpuse of exemplifying how the regression analyses were performed.
The code is also available as an ipython notebook at https://gist.github.com/inti4digbi/a7de296912342c7a6e46505f51fc2875

Import relevant packages which are required to run the exampel regression analyses

```
import pandas as pd
from io import StringIO

import statsmodels.api as sm
from statsmodels.stats.weightstats import ttest_ind
from statsmodels.formula.api import ols, glm
```

Define test data

```
REG_TEST_DATA =
StringIO("""Anxiety_or_depression_T0,Anxiety_severity_T0,Anxiety_severity_T1,BMI_T0,FGID,Gen
der,Age,Weight_loss_group,PC1,PC2,PC3,PC4,PC5,Prevotella
0.0,nan,nan,38.16920425,No,M,63,No
change,0.00725598,0.011931,-0.00560676,0.0108347,0.0149738,nan
1.0,nan,nan,33.90732323,No,M,41,3-5% weight
loss,0.00490136,0.0110828,-0.0108222,-0.000374241,0.0174608,nan
1.0,5.0,2.0,52.07407407,Yes,F,35,3-5% weight
loss,0.0051837,-0.00808046,0.0376884,-0.00370137,0.00651243,nan
0.0,nan,nan,36.15074326,Yes,F,56,3-5% weight
loss,0.00664131,0.0110353,-0.00329147,0.00663005,0.0172224,nan
0.0,2.0,1.0,34.0146135,Yes,F,40,No
change,0.00536844,0.00665504,0.00454055,0.0020724,-0.0193172,nan
1.0,nan,nan,33.85059111,Yes,M,34,5-10% weight
loss,0.00673543,0.0108313,-0.00183264,0.00521545,-0.00237056,1.02957683
1.0,5.0,3.0,29.66161332,Yes,F,39,3-5% weight
loss,0.00612808,0.0119427,-0.00762238,0.0115234,0.0156393,nan
0.0,nan,nan,39.68363265,No,M,53,3-5% weight
loss,0.0067371,-0.00802224,0.0374272,-0.00420121,0.00827515,nan
1.0,nan,nan,31.15588309,Yes,F,56,No
change,0.00651708,0.0108751,-0.00285501,0.0016073,-0.0222636,nan
1.0,3.0,1.0,26.89461505,Yes,F,34,No
change,-0.0257686,0.000625866,-0.000762281,0.00597567,0.0230456,nan
0.0,2.0,1.0,29.1183432,Yes,F,62,5-10% weight
loss,0.00597176,0.0141191,-0.00622036,0.00421402,0.0174668,nan
0.0,nan,nan,32.94532544,Yes,F,48,3-5% weight
```

```
loss,-0.018746,0.00315972,-0.00167929,0.00595569,0.00258426,7.67188119
0.0,nan,nan,41.20856934,Yes,F,40,More than 10% weight
loss,0.00731004,0.0103546,-0.00507988,0.00902664,0.0169948,nan
1.0,nan,nan,31.23824638,No,M,52,No
change,0.00627005,0.0118793,-0.00113265,0.00291227,-0.020415,nan
1.0,3.0,4.0,33.33009324,Yes,F,25,No
change,-0.0096476,0.00608991,-0.00137241,0.0070808,0.0196938,0.87149749
0.0,4.0,3.0,35.76691837,Yes,M,71,5-10% weight
loss,0.0076972,0.0108953,-0.00455194,0.00890042,-0.00326519,nan
0.0,4.0,2.0,40.99219467,Yes,F,39,5-10% weight
loss,0.00761375,0.0109477,-0.00412606,0.00889951,0.0164429,1.08568097
1.0,nan,nan,39.17272222,No,F,40,3-5% weight
loss,-0.0160212,0.00481064,-0.00116674,0.00549903,0.00112346,nan
0.0,5.0,2.0,52.85950255,Yes,F,47,5-10% weight
loss,0.00732507,0.0103358,-0.0045787,0.0039646,-0.00160394,1.25029508
1.0,nan,nan,25.25808284,Yes,M,38,Weight
Gain,0.00629647,-0.0263979,-0.0141875,0.0115217,0.0113855,nan
0.0,nan,nan,37.76446281,Yes,F,54,5-10% weight
loss,0.00754141,0.0125571,-0.00572696,0.0107766,0.0158597,6.87601126
0.0,1.0,3.0,36.02783559,Yes,F,35,3-5% weight
loss,0.00681648,0.0100985,-0.00304681,0.00510866,-0.00304847,3.00252364
1.0,nan,nan,33.00461426,Yes,F,31,More than 10% weight
loss,0.00586998,-0.0272053,-0.0041995,0.0148156,0.00258448,nan
1.0,nan,nan,28.69387755,Yes,F,41,3-5% weight
loss,0.0064213,-0.00574985,0.0297041,-0.000609284,0.0142106,1.93922297
0.0,3.0,2.0,35.92519492,Yes,F,52,5-10% weight
loss,0.00707552,0.00998475,-0.00470972,0.0136878,-0.00744601,0.19339674
1.0,nan,nan,30.99647266,Yes,F,39,5-10% weight
loss,0.00756116,-0.0044101,0.027912,-0.000262882,0.0139352,nan
1.0,nan,nan,32.13714286,No,F,36,5-10% weight
loss,0.00716032,0.000710168,0.018078,0.00444047,0.00404785,nan
0.0,4.0,4.0,32.23633157,Yes,F,32,No
change,0.00634036,0.00267498,0.0230197,-0.00785331,-0.0146848,nan
1.0,nan,nan,46.65994539,Yes,M,36,3-5% weight
loss,0.00760982,0.00931582,0.00288382,0.00600848,0.0166218,nan
1.0,nan,nan,26.56840514,No,F,41,3-5% weight
loss,0.0045291,0.010424,-0.00281774,0.00775594,0.0173857,0.08621969
0.0,nan,nan,27.63256836,Yes,F,40,5-10% weight
loss,0.00453186,-0.00676449,0.0377597,0.000589447,-0.0131775,nan
0.0,nan,nan,42.4295858,No,F,62,More than 10% weight
loss,0.00693492,0.0108041,-0.0028717,0.0066426,-0.00280189,0.96321384
0.0,4.0,1.0,40.88877551,Yes,M,57,5-10% weight
loss,0.00491645,0.00112779,0.0199133,0.000168061,-0.01779,nan
1.0,2.0,1.0,43.21793903,Yes,F,42,5-10% weight
loss,0.00681786,-0.00820915,0.0352405,-0.000495864,0.00620988,6.26029768
1.0,nan,nan,32.8905877,Yes,F,42,5-10% weight
loss,-0.00938019,0.00608578,-0.00227439,0.00368649,-0.0172543,nan
0.0,4.0,2.0,36.70073529,Yes,M,57,3-5% weight
loss,0.00776907,0.0114608,-0.00422116,0.00695537,-0.00220234,nan
0.0,5.0,1.0,31.14761249,No,F,55,5-10% weight
loss,0.00596804,0.0109354,-0.00624086,0.0093805,-0.00268608,nan
0.0,3.0,4.0,37.30204082,Yes,M,62,3-5% weight
```

```
loss,0.0066283,0.00382408,0.0142925,0.000881076,0.00170104,nan
1.0,2.0,1.0,68.12319002,Yes,F,46,No
change,0.00627048,0.0105752,-0.00358,3.49e-05,-0.0194417,nan
1.0,4.0,2.0,35.53081381,Yes,F,38,5-10% weight
loss,0.00419819,0.000620216,0.0117013,0.00536249,0.00104539,nan
1.0,nan,nan,31.73065073,Yes,M,69,3-5% weight
loss,0.00508832,0.000360711,0.0158874,-0.000326743,-0.0139891,7.66875768
1.0,4.0,4.0,25.75084655,Yes,F,32,5-10% weight
loss,0.00716649,0.0109656,-0.00650087,0.00455056,-0.00072865,6.69670297
1.0,3.0,2.0,36.17622348,No,F,33,5-10% weight
loss,-0.0253052,-0.000468775,0.00185719,0.000215523,-0.0144186,nan
0.0,1.0,1.0,29.45550517,Yes,F,59,Weight
Gain,0.00535775,0.00520233,0.0036174,0.00525708,-0.000567761,nan
1.0,nan,nan,33.75463138,No,M,35,5-10% weight
loss,0.00705132,0.0120216,-0.00326211,0.013719,0.0155479,1.42295409
0.0,nan,nan,37.44985352,No,F,60,5-10% weight
loss,-0.0214,8.11e-05,-0.00160536,0.00239025,-0.015761,8.52441262
1.0,nan,nan,32.71646079,Yes,F,39,5-10% weight
loss,0.00573722,0.0089118,0.00647032,0.0062361,0.0203629,6.51686166
0.0,3.0,1.0,35.73324932,Yes,F,43,3-5% weight
loss,-0.0229785,0.00154913,0.00155057,0.00179424,0.00531782,7.81087945
1.0,1.0,1.0,45.80589849,Yes,F,64,3-5% weight
loss,0.00720483,0.00808261,-0.00652149,0.00322679,-0.0208516,nan
0.0,nan,nan,37.30204082,No,M,47,5-10% weight
loss,0.00588777,0.0114043,-0.00559319,0.00820441,0.0169756,nan
1.0,2.0,2.0,25.96795918,Yes,M,52,3-5% weight
loss,0.00806972,0.0104906,-0.00406348,0.0149543,0.0152528,nan
1.0,3.0,3.0,24.36357908,Yes,F,35,5-10% weight
loss,0.00670908,0.0124231,-0.00426958,0.00862894,0.0141588,8.99902559
1.0,nan,nan,32.23633157,Yes,F,51,5-10% weight
loss,0.00459661,0.00659912,-0.00388536,-0.00369747,0.0218908,nan
1.0,nan,nan,32.26063711,Yes,F,67,5-10% weight
loss,0.00815065,0.0114903,-0.0020656,0.00749447,0.0160502,nan
0.0,0.0,0.0,33.63964844,Yes,F,49,5-10% weight
loss,0.00714443,0.0127913,-0.0052784,0.0117562,0.0131353,nan
0.0,nan,nan,31.28142012,Yes,F,64,5-10% weight
loss,0.00720114,0.0114087,-0.00469935,0.00803288,-0.00285222,nan
0.0,nan,nan,32.04099896,No,F,66,5-10% weight
loss,0.00635948,0.00852042,-0.00301261,-0.00622189,-0.0182942,3.62342659
1.0,nan,nan,33.19491124,Yes,F,42,5-10% weight
loss,-0.0252052,0.0012571,0.000584454,0.000511002,0.00551512,5.2794808
1.0,nan,nan,38.0813807,No,M,29,5-10% weight
loss,0.00595879,-0.00790701,0.0372495,-0.00384218,-0.00343697,5.16083636
0.0,3.0,3.0,37.58520829,Yes,F,39,No
change,0.00688308,0.0110842,-0.00189705,0.0101684,0.00746116,7.81037621
0.0,nan,nan,28.14362529,Yes,F,45,3-5% weight
loss,0.00658011,0.0126102,-0.00278584,0.00820449,-0.00353797,nan
1.0,2.0,2.0,42.50944822,No,F,43,5-10% weight
loss,-0.017762,0.00425695,-0.00326997,0.00606172,0.00270497,nan
0.0,nan,nan,30.26805556,Yes,F,30,5-10% weight
loss,0.00670982,-0.0278024,-0.00992026,0.0136681,0.00228329,nan
0.0,5.0,2.0,30.56662075,Yes,F,39,3-5% weight
```

```
loss,0.00617583,0.0117574,-0.00433241,0.0123358,0.0165815,nan
0.0,nan,nan,42.79564497,Yes,F,37,No
change,-0.021181,0.00214577,-0.000950319,0.00172204,-0.0147312,nan
1.0,3.0,3.0,35.69864724,Yes,F,46,5-10% weight
loss,0.00766935,0.0119721,-0.00669481,0.00600627,0.0135873,nan
1.0,nan,nan,26.44441925,Yes,F,62,5-10% weight
loss,0.00757264,0.00347171,0.0144335,-0.00111643,0.000847386,nan
0.0,4.0,4.0,63.40500686,Yes,F,32,5-10% weight
loss,0.00329483,0.00606728,-0.000371508,0.00671881,-0.00129631,nan
0.0,nan,nan,33.9367095,No,F,38,More than 10% weight
loss,0.00738878,-0.012294,-0.00789228,0.0143259,0.0203612,nan
0.0,5.0,5.0,41.62383472,Yes,F,48,3-5% weight
loss,0.00695757,0.0130414,-0.00163012,0.00931508,0.0167581,nan
1.0,nan,nan,39.92383218,Yes,F,52,3-5% weight
loss,-0.0195396,0.00198801,-0.00410494,0.00488312,0.00400968,nan
0.0,4.0,2.0,42.66042388,Yes,F,29,3-5% weight
loss,0.00529847,0.0109063,-0.00489909,0.00059693,-0.0196203,nan
1.0,nan,nan,31.47038567,No,M,46,5-10% weight
loss,0.00764745,0.0132249,-0.00498605,0.00813913,0.0174458,nan
0.0,3.0,3.0,49.54568411,Yes,F,53,3-5% weight
loss,0.00603731,0.00991603,-0.00836552,0.00848246,0.0166113,3.13318666
1.0,5.0,2.0,36.57648283,Yes,F,33,5-10% weight
loss,0.00720594,0.0122705,-0.00522557,0.0089048,0.016467,nan
0.0,5.0,3.0,43.5361996,Yes,F,33,3-5% weight
loss,0.00595817,0.012328,-0.00348677,-0.00103921,0.0204225,6.00612127
0.0,4.0,2.0,34.85950413,Yes,F,37,5-10% weight
loss,0.00763804,0.0108933,-0.00114017,0.007057,-0.00253233,1.67905175
0.0,nan,nan,36.80218311,Yes,M,75,3-5% weight
loss,0.00744997,0.0111731,-0.00447924,0.0076202,-0.00365803,6.72486764
1.0,1.0,1.0,28.5885737,Yes,M,42,5-10% weight
loss,0.0068647,0.0109222,-0.00646639,0.00620923,0.0090966,6.60091342
1.0,nan,nan,49.41798817,No,F,58,3-5% weight
loss,0.00649095,0.0120737,-0.00246991,0.00238451,-0.0211186,4.51206158
0.0,2.0,2.0,28.28639053,Yes,F,62,No
change,0.00551231,0.0117025,-0.00465282,-0.00578998,0.00194847,nan
1.0,nan,nan,30.5159668,Yes,F,30,5-10% weight
loss,-0.00545305,-0.00042258,0.010537,0.0104897,0.0110499,nan
0.0,3.0,0.0,36.35702393,Yes,F,45,5-10% weight
loss,0.00461302,0.00367953,0.0133496,-0.00471989,-0.0165193,1.74433183
0.0,nan,nan,33.37474747,Yes,F,53,5-10% weight
loss,0.0075061,0.0110462,-0.00103515,0.00711876,-0.0115442,nan
1.0,nan,nan,45.99517906,Yes,F,26,No
change,0.00784286,0.0111204,-0.00412017,0.00948871,0.00686843,nan
0.0,nan,nan,29.17724609,Yes,F,64,5-10% weight
loss,0.00733613,0.0116072,-0.00118551,0.0056265,-0.0238152,1.42529658
1.0,nan,nan,33.47619048,Yes,F,54,5-10% weight
loss,0.00810449,0.0109996,-0.000988711,0.00607869,-0.0035805,nan
1.0,nan,nan,32.58416213,Yes,M,44,3-5% weight
loss,0.00460571,0.0105057,-0.00808624,0.00335122,0.0187345,nan
0.0,nan,nan,34.04394667,Yes,M,53,5-10% weight
loss,0.00754883,0.0120734,0.00408531,0.0145862,0.0160571,nan
0.0,nan,nan,31.09001041,Yes,F,45,5-10% weight
```

```
loss,0.00558431,0.00378396,0.0193797,0.00255358,0.0178492,nan
1.0,5.0,3.0,27.95360947,Yes,F,60,3-5% weight
loss,0.00735874,0.0109503,-0.00296784,0.0120738,0.0172668,nan
0.0,5.0,2.0,30.54136316,No,F,25,5-10% weight
loss,0.00582421,-0.000603133,0.0180678,0.000656941,-0.0174494,nan
1.0,5.0,2.0,26.70083247,Yes,F,32,5-10% weight
loss,0.00712087,0.000133682,0.0184023,0.0020018,0.0124178,4.3313328
0.0,2.0,1.0,34.4531076,Yes,M,57,5-10% weight
loss,0.00633801,0.0118428,-0.0036919,0.00564229,-0.0209723,0.67243573
1.0,3.0,2.0,30.41736092,Yes,F,47,5-10% weight
loss,0.00608428,-0.000549406,0.0141993,0.0071771,0.0198441,5.32162542
0.0,5.0,4.0,36.33236801,Yes,F,42,No
change,0.00852392,0.011355,-0.00467047,0.011609,0.0154599,2.30983234
0.0,nan,nan,33.01891892,Yes,M,60,No
change,0.00784928,0.0110815,-0.00543131,0.00727993,0.00821887,nan
1.0,nan,nan,31.58007813,Yes,F,66,3-5% weight
loss,0.00460129,0.00882222,0.0023076,0.0079829,-0.00141017,nan
0.0,2.0,1.0,31.40844727,Yes,F,49,3-5% weight
loss,0.0072,-5.5e-06,0.0144014,0.00341908,0.0222027,nan
1.0,1.0,1.0,32.91883455,Yes,F,58,5-10% weight
loss,0.00749378,0.0114752,-0.00235303,0.00641545,-0.00327197,7.17001873
0.0,1.0,1.0,41.65693918,Yes,F,41,3-5% weight
loss,0.00717049,0.00964957,-0.00392485,0.00640947,-0.00198731,nan
0.0,3.0,3.0,34.19901145,Yes,F,53,3-5% weight
loss,0.0032005,-0.000579554,0.0206616,0.000481465,0.000803068,nan
0.0,nan,nan,44.10473616,Yes,F,44,5-10% weight
loss,0.00765526,0.0101344,-0.00475659,0.00790976,0.0175317,nan
0.0,3.0,1.0,28.12608131,Yes,F,33,5-10% weight
loss,0.00636037,0.013556,-0.00422875,0.00848175,0.0165657,7.4298747
0.0,nan,nan,30.32190427,Yes,F,57,5-10% weight
loss,0.00734234,0.011599,0.000345794,0.0126538,0.0172373,6.97118559
1.0,nan,nan,35.60166289,No,F,40,3-5% weight
loss,0.00707629,0.0103195,-0.00116865,0.00481946,-0.00146859,nan
1.0,3.0,1.0,25.69416823,Yes,F,57,5-10% weight
loss,0.00680538,-0.00390671,0.0276839,-0.000206348,0.0168031,nan
1.0,nan,nan,47.73744379,Yes,F,52,3-5% weight
loss,-0.0274074,0.00139231,-0.00210658,0.00409492,0.0210021,1.51106702
1.0,nan,nan,29.46919192,Yes,F,48,5-10% weight
loss,0.00675488,-0.00237772,0.02613,-0.00507975,-0.013867,nan
1.0,3.0,3.0,33.44723183,Yes,F,29,5-10% weight
loss,0.00709261,0.0116694,-0.00642275,0.00468334,-0.0235001,0.52083548
1.0,nan,nan,32.10403208,Yes,F,54,5-10% weight
loss,-0.0266331,0.00183275,0.000722567,0.00329852,-0.013857,nan
0.0,nan,nan,29.94576844,Yes,M,42,3-5% weight
loss,0.00655059,0.012083,-0.00117196,-0.000772653,-0.020757,nan
0.0,nan,nan,32.76769967,No,F,45,5-10% weight
loss,0.00684996,0.0120552,-0.00181324,0.00365465,0.0163204,1.54291563
0.0,2.0,3.0,36.25867883,Yes,M,64,5-10% weight
loss,0.00655333,0.00783513,-0.00296177,-0.000823423,-0.0201485,1.19304387
1.0,5.0,3.0,31.47038567,Yes,F,35,No
change,0.00788243,0.0122938,-0.00290795,0.00853109,-0.00361071,nan
0.0,4.0,3.0,33.43369141,Yes,F,26,No
```

```
change,-0.00462658,0.00535458,0.00399,7.45e-06,0.00137462,1.25301035
0.0,nan,nan,31.32263184,Yes,F,50,5-10% weight
loss,0.00341356,-0.00418624,0.0236866,0.00160968,-0.00560475,nan
0.0,nan,nan,26.08789063,Yes,F,68,3-5% weight
loss,0.00653026,0.0115857,-0.00443127,0.00658262,-0.00024264,7.39378384
0.0,4.0,4.0,41.47635445,Yes,F,45,No
change,-0.0123054,0.00528363,0.0011308,0.00665375,-0.00628577,nan
0.0,0.0,0.0,34.32617188,Yes,F,32,3-5% weight
loss,0.00745513,0.0104056,-0.00212202,0.000552509,-0.0200465,nan
0.0,2.0,2.0,35.69921875,Yes,F,56,3-5% weight
loss,-0.0253389,0.000542964,0.00051534,0.00208011,-0.012199,nan
0.0,nan,nan,35.10840237,Yes,F,33,5-10% weight
loss,0.00725837,-0.00767914,0.0409734,-0.00609862,-0.00152259,1.95371954
0.0,nan,nan,30.17559834,No,F,71,3-5% weight
loss,0.00601902,0.0107269,-0.004611,0.00547875,-0.000484204,nan
1.0,2.0,2.0,25.42065557,Yes,F,52,No
change,0.0074985,0.00637578,0.00588365,0.00292934,-0.00208827,nan
0.0,nan,nan,27.43236212,Yes,M,27,5-10% weight
loss,0.00594365,-0.0295533,-0.00787649,0.0190897,0.000909668,nan
0.0,nan,nan,29.95029586,Yes,F,57,5-10% weight
loss,0.00772757,0.00991361,-0.00277415,0.00894206,0.016431,nan
0.0,nan,nan,30.51215278,No,M,72,No
change,0.00887728,0.0105139,-0.00123459,0.0126252,0.0146713,4.92001892
0.0,nan,nan,31.79082201,No,M,51,More than 10% weight
loss,0.00740222,0.0122077,-0.00488861,0.00400682,-0.00172446,2.30760129
1.0,nan,nan,28.38335068,Yes,F,60,5-10% weight
loss,0.00644891,0.0110891,-0.00321288,0.00796193,-0.0026252,nan
1.0,4.0,3.0,32.55515243,Yes,F,53,5-10% weight
loss,-0.0206776,0.00205716,0.00274696,0.00665548,0.00120271,nan
1.0,nan,nan,26.46547852,Yes,M,56,5-10% weight
loss,0.00697616,-0.0275822,-0.00469098,0.013686,0.00119614,1.88721658
1.0,3.0,1.0,38.79733701,Yes,F,50,5-10% weight
loss,0.00569922,0.00227929,0.0124,6.36e-06,-0.00557303,nan
0.0,5.0,3.0,27.27433425,Yes,F,64,5-10% weight
loss,0.00711083,0.0117257,-0.0073638,0.00922862,-0.00339363,4.32159136
1.0,3.0,3.0,28.12,Yes,F,38,5-10% weight
loss,0.00693099,0.0105439,0.00177255,0.00505865,-0.0220011,nan
0.0,nan,nan,26.07202365,Yes,F,35,5-10% weight
loss,0.00608818,0.00739298,0.00935404,0.00154414,-0.0187236,1.95454536
0.0,nan,nan,40.99219467,Yes,F,46,More than 10% weight
loss,0.00743038,0.0107959,-0.00494163,0.00337096,-0.0206922,nan
1.0,nan,nan,43.8917794,No,F,61,3-5% weight
loss,0.0057848,-0.00332297,0.028419,-0.00403001,0.0154676,nan
0.0,4.0,4.0,48.48275862,Yes,F,36,5-10% weight
loss,0.00738663,0.0108882,-0.00330357,0.00490995,-0.00223366,7.97658061
1.0,5.0,4.0,25.8244898,Yes,F,32,More than 10% weight
loss,0.00642768,0.00868011,-0.00195761,-0.000863052,-0.0199855,nan
0.0,nan,nan,28.6191716,Yes,F,69,5-10% weight
loss,0.00675112,-0.0277707,-0.0034721,0.00706998,0.00435771,nan
0.0,nan,nan,27.43572084,Yes,F,39,3-5% weight
loss,0.00261016,0.00850745,-0.00801406,0.00241986,0.0186188,7.03139209
1.0,3.0,0.0,51.8329904,Yes,F,67,3-5% weight
```

```
loss,0.00739228,0.0112922,0.000566835,0.00698178,-0.00234993,6.93439335
0.0,4.0,3.0,38.45842882,Yes,F,38,3-5% weight
loss,0.00618456,0.0101188,-0.00402899,0.00308014,-0.00196695,nan
1.0,nan,nan,29.75495656,Yes,F,52,3-5% weight
loss,0.00707523,0.011543,-0.00229843,0.00155051,-0.0214891,nan
0.0,2.0,1.0,30.84591837,Yes,M,41,5-10% weight
loss,0.00732629,0.0124124,-0.00601684,0.00665259,-0.00364452,nan
1.0,4.0,1.0,36.69946939,Yes,M,48,5-10% weight
loss,0.00517858,-0.000762131,0.0206,4.08e-05,0.0043946,nan
0.0,nan,nan,39.64672852,Yes,F,56,5-10% weight
loss,0.0073382,0.010585,-0.00655645,0.00631462,-0.00212091,nan
0.0,nan,nan,28.3190918,Yes,F,67,No
change,0.00677754,-0.00867294,-0.00954732,0.00575992,0.0014477,nan
0.0,nan,nan,35.2747929,Yes,F,62,3-5% weight
loss,0.00771119,0.011,-3.28e-05,0.0081118,0.0168319,nan
0.0,nan,nan,41.54873469,No,F,53,5-10% weight
loss,0.00696551,0.0111385,-0.000407163,0.00148971,-0.021798,5.47209811
0.0,nan,nan,43.15397805,Yes,F,57,5-10% weight
loss,0.00647643,0.0107572,-0.00423085,0.00548675,-0.00221848,6.22453163
1.0,nan,nan,27.43236212,Yes,F,47,5-10% weight
loss,0.00601073,-0.00572842,0.0377754,-0.00692746,-0.00404517,nan
1.0,5.0,0.0,33.46801758,No,F,49,5-10% weight
loss,0.00695992,0.00979904,-0.000127743,0.00326489,-0.0216061,4.79332492
1.0,nan,nan,39.61240234,Yes,F,41,3-5% weight
loss,0.00594087,-0.00142876,0.020434,-0.000996862,-0.0167819,5.50687953
1.0,nan,nan,35.09800296,Yes,F,34,5-10% weight
loss,0.00746459,0.0115743,-0.00301941,0.00444225,0.013217,nan
0.0,5.0,2.0,29.04958678,Yes,F,71,5-10% weight
loss,0.00688342,0.012158,-0.0042506,0.00771024,-0.00373251,3.66794752
0.0,2.0,0.0,36.84152179,Yes,F,64,5-10% weight
loss,0.00480051,0.0107409,-0.000862866,0.00196639,-0.00133531,-0.0303899
1.0,nan,nan,41.79548157,No,M,58,5-10% weight
loss,0.00668504,0.00948039,-0.00784182,-0.000122547,0.000393809,nan
0.0,3.0,3.0,33.29638672,Yes,F,53,5-10% weight
loss,0.00690692,0.010184,-0.00361826,0.00824834,-0.00191782,6.68982079
0.0,nan,nan,47.71685563,No,F,50,5-10% weight
loss,-0.015725,0.00516298,-0.000297062,-0.00428718,-0.0171869,7.23629651
1.0,nan,nan,26.57560811,Yes,M,74,3-5% weight
loss,0.00884632,0.0105255,-0.00805014,0.00876928,0.01483,nan
1.0,nan,nan,33.29638672,No,F,54,3-5% weight
loss,0.00571689,0.00166253,0.00868375,-0.00241693,-0.00798662,nan
0.0,nan,nan,26.62248521,Yes,F,78,3-5% weight
loss,0.00726271,0.00916557,-0.00575919,0.0078159,0.0150718,nan
0.0,nan,nan,31.62283737,Yes,M,40,3-5% weight
loss,0.00412274,-0.00187709,0.01928,-0.00066421,0.00294087,6.78958517
1.0,4.0,1.0,31.00819156,Yes,M,34,5-10% weight
loss,0.0041272,-0.00521851,0.0312865,-0.00529176,-0.0118637,7.06563094
1.0,2.0,2.0,28.43236816,Yes,F,61,5-10% weight
loss,0.00418505,-0.0037526,-0.0150436,-0.035699,0.0090345,nan
1.0,4.0,4.0,29.95029586,Yes,M,44,3-5% weight
loss,0.00633308,0.00982798,-0.00333989,0.00911958,0.017376,0.356182
1.0,1.0,1.0,29.53161101,Yes,F,67,3-5% weight
```

```
loss,0.00740104,0.0107791,-0.00186771,0.00317876,0.0188125,2.05576399
0.0,nan,nan,47.9640955,No,F,61,5-10% weight
loss,0.00730843,0.0113445,-0.00235819,0.00437588,-0.00118064,nan
1.0,0.0,0.0,35.29630593,Yes,F,58,5-10% weight
loss,0.00727835,0.0123623,-0.00108782,0.008992,0.00905617,nan
0.0,nan,nan,26.15218522,Yes,F,52,3-5% weight
loss,0.00751093,0.00966165,0.00133162,0.00608622,-0.000179389,nan
0.0,nan,nan,25.37001559,No,F,67,3-5% weight
loss,0.00607623,0.0103041,-0.00486749,0.00425497,-0.0101576,7.42929161
0.0,nan,nan,30.84591837,No,M,57,5-10% weight
loss,0.00674197,-0.028309,-0.00392176,0.00950773,-0.0158594,nan
1.0,nan,nan,32.02555556,Yes,F,33,5-10% weight
loss,0.00444509,0.011508,-0.00686289,0.00694126,0.0159614,nan
1.0,nan,nan,29.75495656,Yes,M,68,3-5% weight
loss,-0.00486539,0.00776976,-0.000592738,0.0108026,0.0192162,nan
1.0,0.0,0.0,53.0359001,Yes,F,50,5-10% weight
loss,0.00752287,0.00983423,0.00106071,0.0065539,0.0174405,nan
0.0,4.0,3.0,33.27810651,Yes,F,51,3-5% weight
loss,0.0072216,0.001923,0.0180591,0.000508279,0.00266381,0.75567051
1.0,nan,nan,28.69387755,No,F,53,5-10% weight
loss,0.0074109,0.0123316,-0.00839978,0.00868003,0.011257,2.69505301
0.0,5.0,0.0,26.43115234,No,F,50,5-10% weight
loss,0.00232565,0.00806241,0.00471574,0.00173165,0.0178546,nan
1.0,nan,nan,32.99795918,Yes,M,68,3-5% weight
loss,0.00711739,0.0127534,-0.00476322,0.00883556,-0.00323371,nan
0.0,3.0,2.0,27.43572084,Yes,F,59,5-10% weight
loss,0.0067912,0.00965017,-0.00328343,0.00744958,-0.000379444,nan
0.0,2.0,0.0,30.56521739,Yes,F,44,5-10% weight
loss,0.00784568,0.0093794,-0.0023744,0.008385,0.0148083,7.06419527
0.0,nan,nan,29.17724609,Yes,F,50,5-10% weight
loss,-0.020288,0.000496192,-0.00304394,0.00306901,-0.0147632,nan
1.0,nan,nan,20.35865449,Yes,F,41,5-10% weight
loss,0.00566258,0.00967897,-0.00335522,-0.00469866,0.00171317,nan
0.0,3.0,2.0,36.7501929,Yes,F,64,5-10% weight
loss,0.00825966,0.0116039,-0.00355642,0.00994755,0.0151569,nan
0.0,nan,nan,36.20575509,Yes,M,42,5-10% weight
loss,0.00675613,0.0097384,-0.0046199,0.0097275,0.01895,0.98700019
1.0,5.0,3.0,47.38306907,No,F,52,3-5% weight
loss,0.00611378,0.012078,-0.0047649,0.00607726,-0.0223363,0.7482195
0.0,5.0,2.0,29.29166667,Yes,F,53,5-10% weight
loss,0.00683578,0.00706036,-0.00652035,-0.00406071,-0.000214662,nan
1.0,nan,nan,31.06518555,No,F,28,3-5% weight
loss,0.00592579,0.0127959,-0.000831065,0.00102289,-0.0194882,nan
1.0,nan,nan,31.78059172,Yes,F,35,5-10% weight
loss,0.00665879,0.0110232,-0.00511109,0.00769137,-0.00280365,0.99667623
0.0,nan,nan,37.3728899,Yes,F,69,5-10% weight
loss,0.00777137,0.0116751,-0.00467988,0.00358516,-0.00297826,1.22158991
0.0,nan,nan,31.35071807,Yes,F,61,5-10% weight
loss,0.00751236,0.012294,-0.00560168,0.0116547,-0.00398999,nan
0.0,4.0,3.0,34.48047619,Yes,F,54,3-5% weight
loss,-0.00781376,0.00158016,0.0100096,0.00173572,0.00463705,nan
1.0,5.0,1.0,30.8248393,Yes,F,35,5-10% weight
```

```
loss,0.00709926,0.0125717,-0.00419296,0.00547769,-0.0033818,nan
0.0,nan,nan,27.1216568,No,F,54,5-10% weight
loss,0.00686563,-0.0275049,-0.00835329,0.0173962,0.00174579,nan
1.0,4.0,4.0,27.25918367,Yes,M,49,3-5% weight
loss,0.00707787,0.00847623,-0.00319136,-0.000807241,-0.000756723,nan
1.0,2.0,2.0,44.14345703,Yes,F,52,5-10% weight
loss,0.00629346,0.0106747,-0.00408812,0.00692054,-0.0027325,nan
1.0,5.0,4.0,36.01915794,Yes,F,39,5-10% weight
loss,0.00686015,0.0126575,-0.0047556,0.00555016,-0.00370429,nan
1.0,3.0,1.0,31.8577135,Yes,F,64,3-5% weight
loss,0.00731171,0.00971552,-0.00220375,0.0121519,0.00908194,nan
1.0,nan,nan,35.10840237,No,F,65,No
change,0.00729024,0.0112579,-0.000605161,0.00629034,0.0166059,3.68286139
0.0,5.0,0.0,65.82022072,Yes,F,38,5-10% weight
loss,0.00247511,-0.00178077,0.0229951,0.00133034,0.024184,nan
1.0,3.0,1.0,28.12608131,Yes,F,63,5-10% weight
loss,0.00705535,0.0109278,-0.00270525,0.00276828,-0.0116035,nan
0.0,3.0,2.0,34.11005917,Yes,M,35,5-10% weight
loss,0.0070613,0.0111136,-0.00569002,0.00465616,-0.00422569,nan
0.0,nan,nan,35.58559126,Yes,F,70,5-10% weight
loss,-0.0277616,0.000897208,0.00237865,0.00514947,-0.00222543,6.62210804
1.0,2.0,2.0,34.9305411,Yes,F,59,5-10% weight
loss,0.00570301,0.0117131,-0.00251731,0.00613911,-0.00269106,5.27731638
1.0,nan,nan,33.65331318,Yes,F,50,3-5% weight
loss,0.00375389,0.00758388,-0.00549016,0.00464869,-0.00034846,1.87014898
0.0,3.0,3.0,32.04179794,Yes,F,49,3-5% weight
loss,0.00730957,0.0110274,-0.00370505,0.00245726,-0.0219603,nan
1.0,4.0,4.0,28.33963215,Yes,F,37,5-10% weight
loss,0.0050881,-0.0102008,-0.00494567,0.00589767,-0.0185962,nan
0.0,nan,nan,29.2677551,Yes,F,54,3-5% weight
loss,0.00627453,0.012855,-0.00342471,0.00576563,0.00593321,1.67857824
0.0,3.0,3.0,35.01269531,Yes,F,51,5-10% weight
loss,0.00704202,0.0105036,-0.000933372,0.0123608,0.0158241,nan
1.0,0.0,0.0,30.66345271,Yes,F,51,3-5% weight
loss,0.00731836,0.0064303,0.00683554,0.00591562,-0.000162321,nan
0.0,5.0,5.0,25.74462891,Yes,F,25,5-10% weight
loss,0.00660348,-0.0271015,-0.0140092,0.0122281,0.00238258,9.23303543
0.0,4.0,4.0,41.56409567,No,F,56,5-10% weight
loss,0.00665615,0.0116562,-0.00510088,0.000311983,-0.00248494,nan
1.0,nan,nan,35.42454019,No,F,43,5-10% weight
loss,0.00441845,0.00305229,0.0105772,0.00319628,0.00101449,nan
1.0,nan,nan,29.1183432,No,F,40,More than 10% weight
loss,0.00630403,-0.0290725,-0.0094745,0.0152495,0.00185801,nan
1.0,5.0,3.0,48.7313703,Yes,M,43,5-10% weight
loss,0.00268229,-0.00861929,0.033721,-0.00185542,0.0269494,nan
1.0,nan,nan,31.24444444,No,F,66,5-10% weight
loss,0.00651142,0.00909058,-0.00411196,0.00287327,-0.0216312,nan
1.0,nan,nan,50.20373871,No,F,59,5-10% weight
loss,0.00732217,0.0112747,-0.00384527,0.00783917,-0.00227693,nan
0.0,nan,nan,28.70583333,Yes,F,61,5-10% weight
loss,0.00688036,-0.0284086,-0.00737653,0.00952331,-0.0154307,2.23459317
1.0,nan,nan,33.12475586,Yes,F,68,3-5% weight
```

```
loss,-0.0273306,0.0001236,-0.00193953,0.000419042,-0.0114545,1.76272717
0.0,5.0,3.0,25.84015963,Yes,F,59,5-10% weight
loss,0.00747848,0.0104836,-0.00478911,0.00682827,-0.00327563,nan
0.0,nan,nan,27.24947971,Yes,F,56,5-10% weight
loss,0.0058913,0.0114437,-0.00323993,0.0031347,-0.00534335,4.99612422
0.0,nan,nan,57.16358418,Yes,F,47,5-10% weight
loss,0.00783685,0.0110311,-0.00127733,0.00540794,-0.0025441,nan
0.0,nan,nan,25.25061224,Yes,M,30,5-10% weight
loss,-2.35e-05,0.00131678,0.0103266,0.000413603,0.00162757,nan
0.0,nan,nan,26.75778547,Yes,F,33,5-10% weight
loss,0.0078085,-0.00633119,0.0267531,0.0010604,-0.00471133,7.31136056
1.0,3.0,3.0,45.34341144,Yes,F,39,No
change,0.00617317,0.00940979,-0.00277977,0.00291275,-0.0207864,nan
0.0,nan,nan,25.88640955,Yes,F,63,5-10% weight
loss,0.0071104,0.0103901,-0.00275499,0.00899948,0.0164546,nan
0.0,nan,nan,36.80218311,Yes,F,56,5-10% weight
loss,0.00564063,-0.000934505,0.0133755,0.00333482,-0.0177583,nan
1.0,nan,nan,26.94833333,No,F,63,No
change,-0.0138889,0.00297268,-0.00175549,0.0048127,0.00265499,nan
1.0,nan,nan,31.74648183,Yes,M,42,5-10% weight
loss,0.00634813,-0.00128241,0.02119,0.00436593,0.0046308,nan
0.0,nan,nan,28.89542144,Yes,F,39,5-10% weight
loss,0.00648169,-0.029809,-0.0111483,0.0129522,-0.01439,nan
0.0,2.0,2.0,50.39214033,Yes,M,29,More than 10% weight
loss,0.00553388,0.0112211,-0.0045968,0.00499888,0.0191168,nan
1.0,2.0,1.0,25.84015963,Yes,F,24,5-10% weight
loss,0.00738727,0.0115767,-0.00357124,0.00562106,-0.0206125,nan
1.0,nan,nan,55.14604592,Yes,F,42,More than 10% weight
loss,0.00185679,-0.00731187,0.0350164,-0.0053393,0.00842807,1.58577371
1.0,nan,nan,28.22861604,Yes,M,67,5-10% weight
loss,0.00572904,0.0119575,-0.00539307,0.0101874,-0.00170517,nan
0.0,nan,nan,44.75905325,Yes,F,58,5-10% weight
loss,0.00674893,0.0100096,-0.00478408,0.00351474,-0.0214764,2.77118465
0.0,nan,nan,38.79138483,Yes,F,69,5-10% weight
loss,0.00560811,-0.0306982,-0.00955016,0.0104097,-0.0143958,nan
1.0,nan,nan,24.69214876,Yes,F,53,3-5% weight
loss,0.00723448,0.00932793,-0.00177304,0.00676846,-0.0238145,nan
0.0,3.0,1.0,46.16870117,Yes,F,30,No
change,0.00731808,0.0130931,-0.00143055,0.00616703,0.0160761,1.06757639
1.0,4.0,4.0,41.00833333,Yes,F,29,5-10% weight
loss,0.0066248,0.00914439,-0.00381106,0.00767449,-0.00350438,nan
0.0,nan,nan,29.2362949,Yes,F,58,5-10% weight
loss,0.00629745,0.0119486,-0.00564135,0.00110619,-0.0209146,nan
0.0,nan,nan,34.56477627,Yes,F,41,5-10% weight
loss,0.00557686,-0.00637023,0.0349809,-0.00153876,0.00578056,nan
1.0,nan,nan,41.94195109,Yes,F,59,5-10% weight
loss,0.00559008,0.0020437,-0.00952189,-0.0226453,-0.0150669,nan
0.0,nan,nan,30.1499645,Yes,F,60,3-5% weight
loss,-0.0161593,0.00325,5.79e-05,-0.000329127,-0.0129074,nan
1.0,2.0,1.0,38.96699421,Yes,F,70,3-5% weight
loss,0.00628652,0.0121157,-0.00209886,0.0121203,0.0158757,2.90949995
0.0,nan,nan,36.01915794,Yes,F,72,5-10% weight
```

```
loss,0.00683925,0.0105932,-0.000209056,0.00738521,-0.0220485,1.18875554
1.0,nan,nan,27.53416667,Yes,F,70,No
change,-0.0205672,-0.00343695,0.0113517,-0.00170188,-0.0121557,2.47732269
1.0,nan,nan,33.31138462,Yes,M,68,5-10% weight
loss,0.00515658,-0.00312415,0.0273496,0.00284471,0.024995,nan
1.0,1.0,1.0,25.95692308,No,F,63,5-10% weight
loss,0.00613772,-0.0282007,-0.00637923,0.0144985,0.00223976,nan
0.0,3.0,2.0,36.76403061,Yes,F,50,5-10% weight
loss,0.00554936,-0.027446,-0.0110395,0.0127633,-0.0160186,nan
0.0,nan,nan,43.04081633,Yes,M,43,5-10% weight
loss,-0.00852975,0.00593326,-0.000207632,0.00583293,0.0193034,nan
0.0,4.0,3.0,27.09267097,Yes,F,29,5-10% weight
loss,0.00606833,0.011451,-0.00402542,0.00505198,-0.00206496,nan
0.0,5.0,2.0,33.56841139,Yes,F,42,5-10% weight
loss,-0.00350204,0.00868895,-0.00534879,0.00250113,-0.00142963,nan
0.0,nan,nan,26.6027832,Yes,F,56,5-10% weight
loss,0.00504202,0.00128768,0.0135757,0.00669347,0.000965901,nan
0.0,5.0,0.0,29.44174649,Yes,F,53,More than 10% weight
loss,0.00498789,-0.00775887,0.0319324,-0.00428683,-0.0101037,4.85986571
0.0,5.0,3.0,32.29178201,Yes,F,34,3-5% weight
loss,0.00705733,-0.00277871,0.0116884,-0.000192397,-0.0162725,6.50479594
0.0,nan,nan,34.66943359,Yes,F,55,5-10% weight
loss,0.00702929,0.0129414,-0.00461688,0.00881171,0.0158779,4.19224938
0.0,nan,nan,38.36823346,Yes,M,45,5-10% weight
loss,0.00312668,0.0061575,-0.000340271,0.0184161,0.00263633,nan
0.0,nan,nan,36.7501929,Yes,M,45,3-5% weight
loss,0.00711184,0.00992343,-0.00282501,0.0111682,-0.00237346,6.52280517
0.0,nan,nan,27.57054156,Yes,M,33,3-5% weight
loss,0.00276607,-0.00323294,0.0213886,-0.00183331,-0.00275357,7.38937369
1.0,4.0,0.0,41.09846154,Yes,F,51,5-10% weight
loss,0.0070957,0.0122385,-0.00371869,0.00997052,-0.00226446,1.92232951
0.0,2.0,1.0,36.27970615,Yes,F,41,5-10% weight
loss,0.00734506,0.0131178,-0.00379449,0.00172099,-0.0223585,nan
0.0,3.0,3.0,34.49780273,Yes,F,45,5-10% weight
loss,0.00686347,0.00990794,-0.00152945,-0.000725157,-0.0195172,nan
1.0,4.0,1.0,43.0977055,Yes,F,38,5-10% weight
loss,-0.0119923,0.00379,-8.87e-05,0.00781989,0.00156177,3.32437393
0.0,3.0,1.0,52.33763265,Yes,F,48,5-10% weight
loss,0.00621924,0.00540207,0.00316239,0.00841327,-0.000578129,nan
1.0,nan,nan,39.02710718,Yes,F,45,5-10% weight
loss,0.003797,-0.00511208,0.025787,-0.00239489,-0.0125898,1.69864433
1.0,nan,nan,44.18092105,Yes,M,50,More than 10% weight
loss,0.00793821,0.0107014,-0.00404039,0.0130584,-0.00360167,nan
0.0,nan,nan,45.04805981,Yes,F,45,5-10% weight
loss,0.00670853,0.0108055,-0.00150611,0.00168549,-0.00468786,0.93089182
1.0,nan,nan,26.30825617,Yes,M,58,3-5% weight
loss,0.00726026,0.0104785,-0.00230114,0.00720731,-0.0215404,nan
0.0,nan,nan,38.10205078,Yes,F,70,5-10% weight
loss,0.00781707,0.00299375,0.020598,0.00302177,0.0019756,2.3328237
0.0,nan,nan,31.96621622,Yes,M,38,5-10% weight
loss,0.0039934,0.00924568,-0.00398541,0.00398866,-0.0206906,7.36784339
1.0,nan,nan,26.62285587,Yes,F,61,5-10% weight
```

```
loss,0.00615708,0.00924,-0.00263411,0.00282501,-0.0184086,2.61611685
0.0,3.0,3.0,35.43793321,Yes,M,36,5-10% weight
loss,0.00706676,0.00846723,-0.00524627,0.00926552,0.015642,nan
0.0,nan,nan,33.47619048,Yes,F,46,5-10% weight
loss,0.00689562,-0.0292844,-0.00859857,0.0161551,0.0212809,nan
0.0,2.0,1.0,28.62837838,Yes,M,72,5-10% weight
loss,0.00676276,0.0135014,-0.00583847,0.00883765,-0.00271292,nan
0.0,nan,nan,36.28234104,Yes,M,59,5-10% weight
loss,0.00791471,0.0107045,-0.00266415,0.00257361,-0.00420883,nan
0.0,nan,nan,26.7794609,Yes,F,55,5-10% weight
loss,0.00650576,0.0121312,-0.00266563,0.00922914,-0.00181464,nan
0.0,nan,nan,42.6718107,Yes,F,39,5-10% weight
loss,0.00747715,0.0113853,0.00012506,0.012672,-0.00336445,nan
1.0,3.0,3.0,36.02783559,Yes,F,32,5-10% weight
loss,0.00635289,0.0115535,-0.00110171,0.0106551,-0.00259109,nan
0.0,4.0,2.0,44.96728516,Yes,F,45,5-10% weight
loss,0.00443459,0.00854399,-0.00140219,0.00283743,-0.0188848,nan
1.0,nan,nan,30.24384757,Yes,F,58,3-5% weight
loss,0.00621829,0.0116082,-0.000626788,0.00555851,-0.00111062,nan
1.0,nan,nan,31.58007813,Yes,F,60,5-10% weight
loss,0.00768051,0.0115692,-0.00571516,0.0107045,0.0152366,nan
0.0,nan,nan,29.78390533,Yes,F,66,5-10% weight
loss,0.00685994,0.0120831,-0.00571967,0.00811601,0.0171443,nan
1.0,nan,nan,33.78305556,Yes,F,63,5-10% weight
loss,0.00645547,0.00986087,-0.00129837,0.00633244,-0.00276033,5.14583566
1.0,3.0,1.0,51.55931122,Yes,M,60,More than 10% weight
loss,0.00750616,0.012983,0.00078556,0.007681,-0.00363219,6.0609191
0.0,nan,nan,43.39506173,Yes,F,67,5-10% weight
loss,0.00723893,0.011683,-0.00480849,0.00870961,-0.00397302,0.14999677
0.0,3.0,1.0,30.44946746,Yes,F,26,3-5% weight
loss,0.00485578,0.00226921,-0.00557954,-0.0194165,-0.0152233,nan
1.0,3.0,3.0,32.48477211,Yes,F,45,5-10% weight
loss,0.0075123,0.0100902,-0.00388389,0.00514027,0.0179808,3.01582548
0.0,nan,nan,25.40136719,Yes,F,48,5-10% weight
loss,0.00732352,0.0120024,-0.00276903,0.00207662,-0.0026099,5.7477041
1.0,nan,nan,25.42077581,Yes,M,40,3-5% weight
loss,0.00715089,0.0124023,-0.000593363,0.00917784,0.0169901,7.1906468
1.0,5.0,1.0,27.97653061,Yes,M,32,5-10% weight
loss,0.00661391,0.0103954,0.00180316,0.00115782,0.0006486,0.95008836
1.0,3.0,0.0,35.09851074,Yes,F,54,5-10% weight
loss,0.00759783,0.0109927,-0.00181726,0.00375681,-0.00288019,6.17586489
0.0,2.0,2.0,30.66345271,Yes,F,56,5-10% weight
loss,0.00665101,0.0117609,-0.00361294,0.00123915,-0.000924347,nan
0.0,nan,nan,50.83677686,Yes,F,61,5-10% weight
loss,0.00622803,0.0113308,-0.00516408,0.0051103,-0.00252909,5.09281978
0.0,nan,nan,34.0022449,No,M,44,5-10% weight
loss,-0.0242454,-0.00170247,0.00769824,-0.000498945,-0.0110825,3.41599474
0.0,nan,nan,34.14081441,Yes,M,49,3-5% weight
loss,0.00728357,0.0110019,-0.00168792,0.00181824,-0.020879,nan
1.0,nan,nan,26.72610796,Yes,M,57,5-10% weight
loss,0.00602883,-0.0283283,-0.00690928,0.0168124,0.00129425,nan
1.0,5.0,3.0,42.75317443,Yes,M,37,3-5% weight
```

```
loss,-0.0244822,0.001305,-0.00110374,0.00719755,0.0205597,nan
0.0,nan,nan,32.10303281,Yes,F,35,No
change,0.000610443,0.00712632,-0.00223687,0.00243525,-0.0181336,0.7257482
0.0,nan,nan,47.4525,Yes,F,27,5-10% weight
loss,0.00687927,-0.00375594,0.0215828,0.00137436,0.0241004,1.49454403
1.0,2.0,0.0,29.04958678,Yes,F,57,3-5% weight
loss,0.00655711,0.0108917,-0.00477967,0.00949962,-0.00234152,nan
0.0,nan,nan,25.23777315,Yes,F,49,3-5% weight
loss,0.00785604,0.00859,-1.52e-05,0.00648952,-0.0211146,4.74327975
0.0,nan,nan,27.43236212,Yes,F,58,3-5% weight
loss,0.00674687,0.00481513,0.0163806,0.000609233,0.00197026,nan
0.0,4.0,4.0,41.15358127,Yes,F,42,3-5% weight
loss,0.0063818,0.00484851,0.00812058,-0.000163276,0.000562374,5.7832026
0.0,4.0,2.0,31.61420118,Yes,F,33,5-10% weight
loss,0.00739413,0.0108236,0.00040615,0.00669417,-0.0230405,nan
1.0,0.0,0.0,32.96288889,Yes,F,59,5-10% weight
loss,0.00721784,0.0102659,-0.00162234,0.0112801,-0.00303946,nan
1.0,3.0,3.0,41.04887543,Yes,F,35,No
change,0.00565014,-0.000135834,-0.00405803,-0.032518,-0.00966224,nan
0.0,2.0,2.0,41.36303711,Yes,F,57,3-5% weight
loss,0.00716956,0.0122214,-0.00525563,0.00748105,0.0169404,nan
0.0,nan,nan,30.66345271,Yes,F,55,5-10% weight
loss,0.00815804,0.0139683,-0.0062191,0.0137707,0.0148767,7.45760512
1.0,nan,nan,32.11771029,Yes,F,47,5-10% weight
loss,0.00321954,0.00871552,0.00108722,-0.000425367,-0.000118801,nan
0.0,nan,nan,30.65861111,Yes,F,57,5-10% weight
loss,0.00646074,-0.00780625,-0.00829937,0.00811,9.29e-05,nan
0.0,5.0,2.0,28.72681359,Yes,F,47,5-10% weight
loss,0.00793086,0.0127021,-0.00532173,0.00704605,-0.00227774,nan
1.0,nan,nan,34.4531076,Yes,F,71,5-10% weight
loss,0.00736773,0.0107749,-0.00256518,0.00412367,0.0185866,4.47485371
0.0,nan,nan,42.96996725,Yes,F,37,5-10% weight
loss,0.00724953,0.0107489,-0.00153653,0.00726685,-0.00231643,nan
0.0,nan,nan,36.79195502,Yes,F,58,5-10% weight
loss,-0.00759679,0.00552459,-0.00315112,0.0072691,0.0127593,4.47207219
0.0,5.0,4.0,45.15372212,Yes,F,41,5-10% weight
loss,0.0061405,-0.00502981,0.028161,-0.00299556,0.00394338,1.14289173
0.0,3.0,2.0,36.95752984,Yes,F,44,5-10% weight
loss,0.00779053,0.012044,-0.00319436,0.00970775,0.0155177,nan
1.0,nan,nan,25.8398307,No,M,27,5-10% weight
loss,0.00547637,-0.026809,-0.0100965,0.0129579,0.00250488,nan
1.0,2.0,2.0,20.98025712,Yes,F,44,No
change,-0.0105389,0.00544083,0.000669812,0.00662013,0.019366,nan
1.0,nan,nan,33.96135266,Yes,F,38,5-10% weight
loss,-0.0227582,0.00179467,-0.00112301,0.00699933,0.0102447,0.38148625
1.0,3.0,1.0,31.32100691,Yes,M,42,5-10% weight
loss,0.00595082,-0.0255772,-0.0109544,0.00272015,-0.0145655,nan
1.0,2.0,2.0,27.79812695,Yes,F,41,No
change,0.00777354,0.010152,-0.00248234,0.0114621,0.0110001,nan
0.0,nan,nan,38.43621302,Yes,M,37,5-10% weight
loss,0.00497391,-0.00247695,0.0219724,-0.00199877,-0.0153146,1.93543006
0.0,nan,nan,36.02783559,Yes,F,41,5-10% weight
```

```
loss,0.00631395,-0.0270813,-0.0109486,0.0134482,0.00274338,nan
0.0,nan,nan,32.81387692,Yes,M,47,5-10% weight
loss,0.00628874,0.0108717,-0.00301236,0.0145315,0.0127932,nan
1.0,nan,nan,31.38364892,No,F,49,3-5% weight
loss,-0.0260756,-0.000387376,-0.000599251,0.00295159,0.00468003,nan
1.0,nan,nan,31.94742704,Yes,F,31,5-10% weight
loss,0.00488842,0.00681563,0.00654999,0.00144122,0.00139685,nan
1.0,2.0,2.0,38.76838235,No,M,41,No
change,0.0078388,0.0127005,-0.00299705,0.00573761,-0.00319376,nan""")
```

Read test data provided as string into a pandas data frame

```
sample_data = pd.read_csv(LOG_REG_TEST_DATA)
sample_data.head()
```

Logistic Model

Use the test data to fit a logistic model with outcomes being anxiety or depression at baseline and multiple predictors.

```
# formula for model
f_log_reg = 'Anxiety_or_depression_T0 ~ 1 + BMI_T0 + FGID + Gender + Age +
Weight_loss_group + PC1 + PC2+ PC3+ PC4+ PC5 '
# define GLM
model_log_reg = glm(f_log_reg,                          # formula
                    data=sample_data,                   # input data
                    family=sm.families.Binomial()) # set Binomial family
to fit a logistic regression. see statsmodels documentation for details
results_log_reg = model_log_reg.fit(cov_type='HC3') # Fit the model using a
HC3 covariance matrix. see article main text for additional details
results_log_reg.summary()                              # summarize the model
fitted
```

Example Output

Generalized Linear Model Regression Results

**Dep. Variable:**	Anxiety_or_depression_T0	**No. Observations:**	328
**Model:**	GLM	**Df Residuals:**	314
**Model Family:**	Binomial	**Df Model:**	13
**Link Function:**	Logit	**Scale:**	1.0000
**Method:**	IRLS	**Log-Likelihood:**	-216.90
**Date:**	Tue, 19 Jul 2022	**Deviance:**	433.80
**Time:**	10:31:44	**Pearson chi2:**	328.
**No. Iterations:**	4	**Pseudo R-squ. (CS):**	0.05275
**Covariance Type:**	HC3		

	**coef**	**std err**	**z**	**P>|z|**	**[0.025**	**0.975]**
**Intercept**	2.2023	0.908	2.426	0.015	0.423	3.982
**FGID[T.Yes]**	-0.5875	0.323	-1.817	0.069	-1.221	0.046
**Gender[T.M]**	0.2200	0.283	0.777	0.437	-0.335	0.775
**Weight_loss_group[T.5-10% weight loss]**	-0.2282	0.278	-0.822	0.411	-0.772	0.316
**Weight_loss_group[T.More than 10% weight loss]**	-0.0127	0.687	-0.019	0.985	-1.360	1.334
**Weight_loss_group[T.No change]**	0.0877	0.450	0.195	0.845	-0.794	0.970
**Weight_loss_group[T.Weight Gain]**	0.0459	1.334	0.034	0.973	-2.568	2.660
**BMI_T0**	-0.0291	0.018	-1.654	0.098	-0.064	0.005
**Age**	-0.0131	0.010	-1.357	0.175	-0.032	0.006
**PC1**	-21.2413	14.369	-1.478	0.139	-49.404	6.922
**PC2**	3.6942	11.698	0.316	0.752	-19.234	26.622
**PC3**	5.6530	11.895	0.475	0.635	-17.662	28.968
**PC4**	-27.5838	21.026	-1.312	0.190	-68.794	13.626
**PC5**	15.8115	9.274	1.705	0.088	-2.366	33.989

Poisson regression

```
f_possion_reg = 'Anxiety_severity_T1 ~ 1 + Prevotella + BMI_T0 + FGID +
Gender + Age + Weight_loss_group + PC1 + PC2 + PC3+ PC4+ PC5 '
```

```
# create a boolean index with:
# - True for individuals with Prevotella abundance not NaN, i.e., read
counts > 0
# - True for individuals that provided a baseline anxiety severity score,
entry is not NaN
smp_idx = pd.notna(sample_data['Prevotella']) *
pd.notna(sample_data['Anxiety_severity_T0'])

model_poi_reg = glm(f_possion_reg,
# formula
                    sample_data,
# input data
                    family=sm.families.Poisson(),
# set Poisson family to fit the regression. see statsmodels documentation
for details
                    offset=sample_data.loc[smp_idx, 'Anxiety_severity_T0'],
# set columns with offset for poisson model. In this case offset is the
score at baseline
                    subset=smp_idx)
# indicate the subset of the data to be analysed

results_poi_reg = model_poi_reg.fit(cov_type='HC3')
# Fit the model using a HC3 covariance matrix. see article main text for
additional details
results_poi_reg.summary()
# summarize the model fitted
```

Example Output

Generalized Linear Model Regression Results

**Dep. Variable:**	Anxiety_severity_T1	**No. Observations:**	51
**Model:**	GLM	**Df Residuals:**	37
**Model Family:**	Poisson	**Df Model:**	13
**Link Function:**	Log	**Scale:**	1.0000
**Method:**	IRLS	**Log-Likelihood:**	-109.52
**Date:**	Tue, 19 Jul 2022	**Deviance:**	103.90
**Time:**	10:32:42	**Pearson chi2:**	198.
**No. Iterations:**	6	**Pseudo R-squ. (CS):**	0.4395
**Covariance Type:**	HC3		

	**coef**	**std err**	**z**	**P>|z|**	**[0.025**	**0.975]**
**Intercept**	-4.6818	1.393	-3.362	0.001	-7.411	-1.953
**FGID[T.Yes]**	1.6743	0.756	2.216	0.027	0.193	3.156
**Gender[T.M]**	0.2509	0.591	0.424	0.671	-0.908	1.410
**Weight_loss_group[T.5-10% weight loss]**	-0.5399	0.381	-1.415	0.157	-1.288	0.208
**Weight_loss_group[T.More than 10% weight loss]**	-1.7188	1.006	-1.708	0.088	-3.691	0.253
**Weight_loss_group[T.No change]**	-0.3469	0.668	-0.519	0.604	-1.657	0.963
**Prevotella**	0.0420	0.058	0.721	0.471	-0.072	0.156
**BMI_T0**	0.0040	0.019	0.217	0.829	-0.032	0.040
**Age**	-0.0131	0.021	-0.625	0.532	-0.054	0.028
**PC1**	-21.1965	21.414	-0.990	0.322	-63.168	20.775
**PC2**	25.8532	19.751	1.309	0.191	-12.859	64.565
**PC3**	5.9148	25.584	0.231	0.817	-44.230	56.059
**PC4**	78.6778	67.113	1.172	0.241	-52.862	210.218
**PC5**	-8.6780	17.796	-0.488	0.626	-43.558	26.202

Supplementary Table Legends

Table S1. Demographic data of participants and survey information used in the study.

Table S2. Summary characteristics of the cohort.

Table S3. Genetic scores used in the study.

For each genetic score used we provide its abbreviation as used on the main text, the number of genetic variants, reference to the original scientific study, and GWAS Catalog identifier when available.

Table S4. Summary characteristics of the cohorts studied for the improvement of anxiety, depression, and insomnia.

We provide mean and standard deviation (i.e. mean(std)) for BMI at T0 and Age, and counts for other variables included in all models. P-values were calculated with a t-test to compare means and a chi-squared test for count tables.

Table S5. Summary statistics of genetic and microbiome factors significantly associated with the study outcomes.

For each of the outcomes, we provide the mean and standard deviation of the genetic scores and microbiome taxa and pathways identified as statistically significant.

Table S6. Statistical associations with improvement in anxiety.

A. Associations with demographic variables. **B.** Associations with genetic scores. **C.** Associations with microbiome genera. **D.** Associations with microbiome functional pathways.

Table S7. Statistical associations with improvement in depression.

A. Associations with demographic variables. **B.** Associations with genetic scores. **C.** Associations with microbiome genera. **D.** Associations with microbiome functional pathways.

Table S8. Statistical associations with improvement in insomnia.

A. Associations with demographic variables. **B**. Associations with genetic scores. **C.** Associations with microbiome genera. **D.** Associations with microbiome functional pathways.

Table S9. Summary characteristics of the cohorts studied at baseline.

We provide mean and standard deviation (i.e. mean(std)) for BMI at T0 and Age, and counts for other variables included in all models. P-values were calculated with a t-test to compare means and a chi-squared test for count tables.

Table S10. Statistical associations with anxiety or depression at baseline.

A. Associations with demographic variables. **B.** Associations with genetic scores. **C.** Associations with microbiome genera. **D.** Associations with microbiome functional pathways.

Table S11. Statistical associations with sleep problems at baseline.

A. Associations with demographic variables. **B.** Associations with genetic scores. **C.** Associations with microbiome genera. **D.** Associations with microbiome functional pathways.

Table S12. Model fit of combined models of demographics, microbiome, and genetic predictors.

For each outcome, we performed a bootstrap analysis with 1001 replications of the models including demographic predictors (D), demographic and microbiome predictors (D+M), demographic and genetic predictors (D+G), and all three sets of predictors (D+M+G). For microbiome and genetic predictors, we included in the models variables identified on univariate analyses with an FDR ≤ 0.15. Model fit is reported as the median, mean and standard deviation of the Cox-Snell pseudo r-squared obtained from 1001 bootstrap replicates.

Table S13. Effect of medications on the gut microbiome.

Statistical associations of self-reported intake of anxiolytics and depression medications on the gut microbiome. The potential confounding effect of the medication on the results of the study is indicated by the association of the interaction between medication and the study outcomes. Panels A through F show multivariate analyses based on Bray-Curtis dissimilarity to test the influence of covariates on the gut microbiome and anxiety or depression at baseline, or sleep issues at baseline. PERMANOVAs were performed with 999 bootstrap iterations. **A.** PERMANOVA using bacterial taxa at the genus level (genera: n=178) to test the influence of covariates on the gut microbiome and anxiety or depression at baseline. **B.** PERMANOVA using bacterial taxa at the genus level (genera: n=178) to test the influence of covariates on the gut microbiome and sleep issues at baseline. **C.** PERMANOVA using bacterial functions (gut-brain modules: n=42) to test the influence of covariates on the gut microbiome and anxiety or depression at baseline. **D.** PERMANOVA using bacterial functions (gut-brain modules: n=42) to test the influence of covariates on the gut microbiome and sleep issues at baseline. **E.** PERMANOVA using only bacterial genera and functions with significant association from the regression models (genera: n=15, and gut-brain modules: n=7) to test the influence of covariates on the gut microbiome and anxiety or depression at baseline. **F.** PERMANOVA using only bacterial genera and functions with significant association from the regression models (genera: n=15, and gut-brain modules: n=7) to test the influence of covariates on the gut microbiome and sleep issues at baseline. **G.** Univariate analysis to test the influence of covariates on the gut microbiome and outcomes of interest. The table describes results from linear regression analyses using bacterial CLR transformed abundances and as covariates gender, age, BMI at T0, and weight loss group (weight Gain > 3%, No Change, Weight loss 3-5%, Weight loss 5-10% and Weight loss +10%) using only bacterial genera and functions with

significant associations to four outcomes, namely anxiety intensity change, depression intensity change, insomnia intensity change, and sleep problems at T0. For each outcome and associated microbiome factor, we report beta and beta se corresponding to the regression coefficients and their standard error, respectively. z and P-value of statistical test evaluating beta ≠ 0. '0.025' and '0.975' correspond to the confidence intervals of the beta. anx_change, dep_change, and ins_change are changes in anxiety, depression, and insomnia from T0 to T1.

Supplementary Figures

Figure S1. Study design and cohort sample size for each outcome.

369 subjects were recruited from the Digbi Health digital care program for whom we obtained microbiome (n = 344) and genomic SNP (n = 348) data and selected for analyses the subset of 328 subjects with both datasets (n = 328). Data were analyzed to identify gut microbiome and genetic scores associated with anxiety or depression and sleep problems at baseline (T0) and improvement in anxiety, depression, and insomnia at follow-up (T1), when subjects reached at least 2% body weight loss. For analyses at T1, we excluded individuals who had higher scores at T1 than at T0, and those improved 4 or 5 scale points, leading to sample sizes for anxiety, depression, and insomnia intensity changes of 135,135, and 154, respectively.

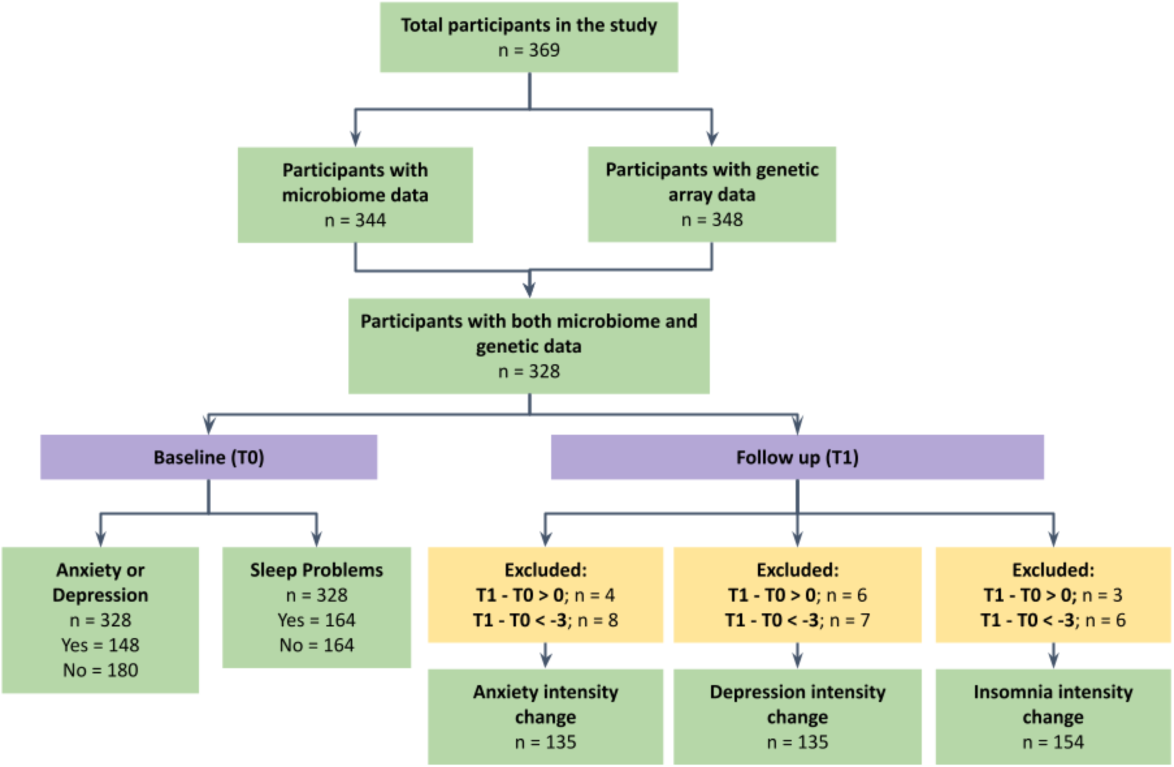

Figure S2. Changes in symptom intensity after the intervention.

The bar plots illustrate the percent of users who self-reported symptom intensity changes in anxiety, depression, and insomnia at follow-up (T1) compared to baseline (T0), on a scale from 0 to 5.

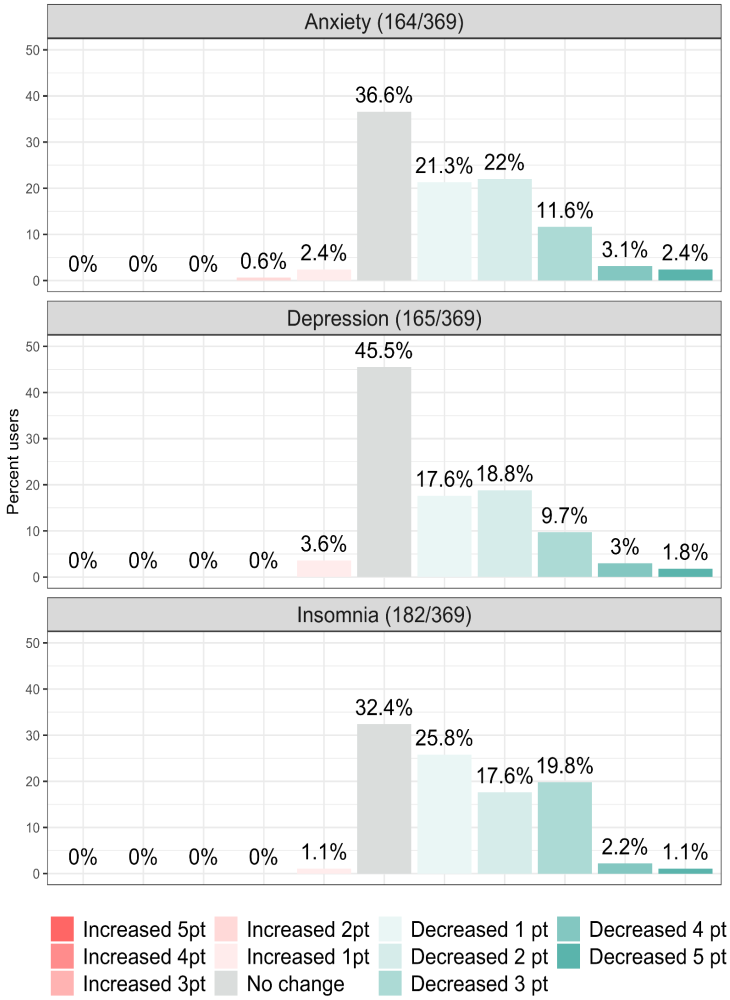

Figure S3. Boxplot of microbiome features associated with improvement on anxiety.

Each panel provides violin plots (sky blue fill), box plots (pink fill), and horizontally jittered points presenting CLR transformed abundances of bacterial genera and functional pathways (y-axis) for each level of improvement of intensity on the outcome measured at T1 - T0 (x-axis). Panel titles display the name of the respective microbiome feature.

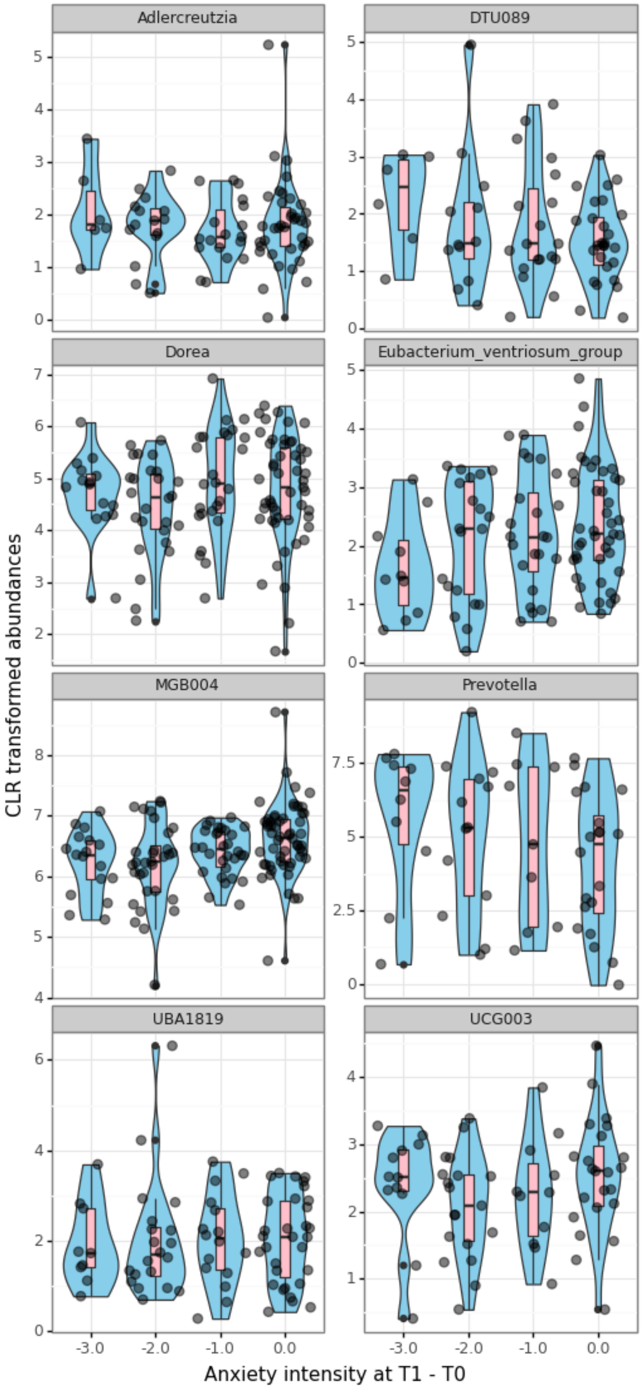

Figure S4. Boxplot of genetic scores associated with improvement on anxiety. +

Each panel provides violin plots (sky blue fill), box plots (pink fill), and horizontally jittered points presenting genetic score values (y-axis) for each level of improvement of intensity on the outcome measured at T1 - T0 (x-axis). Panel titles display the name of the respective genetic score.

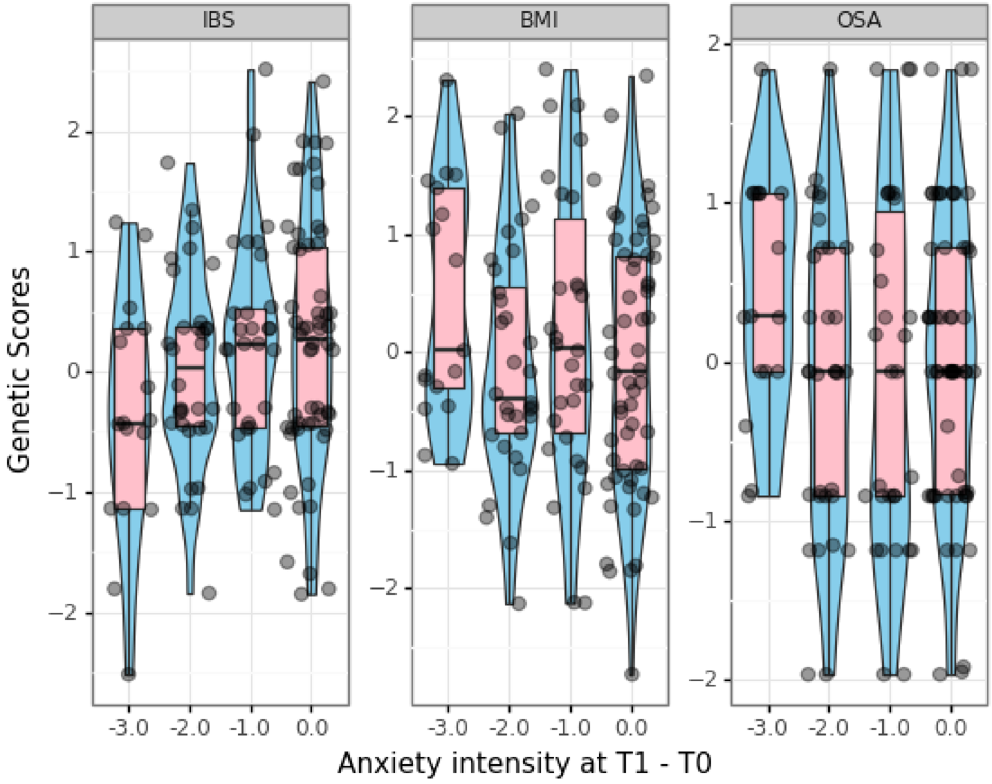

Figure S5. Boxplot of microbiome features associated with improvement on depression.

Each panel provides violin plots (sky blue fill), box plots (pink fill), and horizontally jittered points presenting CLR transformed abundances of bacterial genera and functional pathways (y-axis) for each level of improvement of intensity on the outcome measured at T1 - T0 (x-axis). Panel titles display the name of the respective microbiome feature.

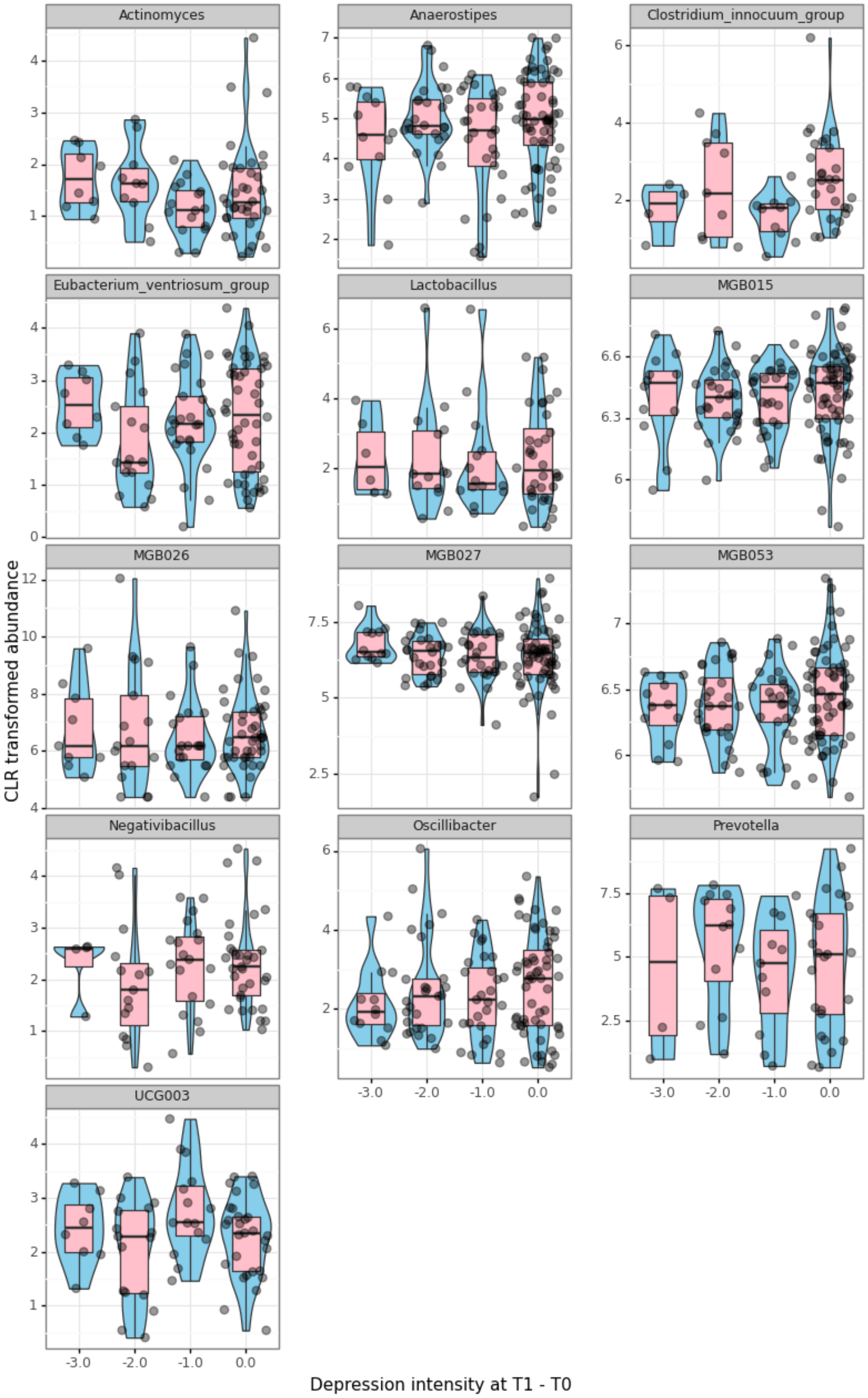

Figure S6. Boxplot of genetic scores associated with improvement on depression.

Each panel provides violin plots (sky blue fill), box plots (pink fill), and horizontally jittered points presenting genetic score values (y-axis) for each level of improvement of intensity on the outcome measured at T1 - T0 (x-axis). Panel titles display the name of the respective genetic score.

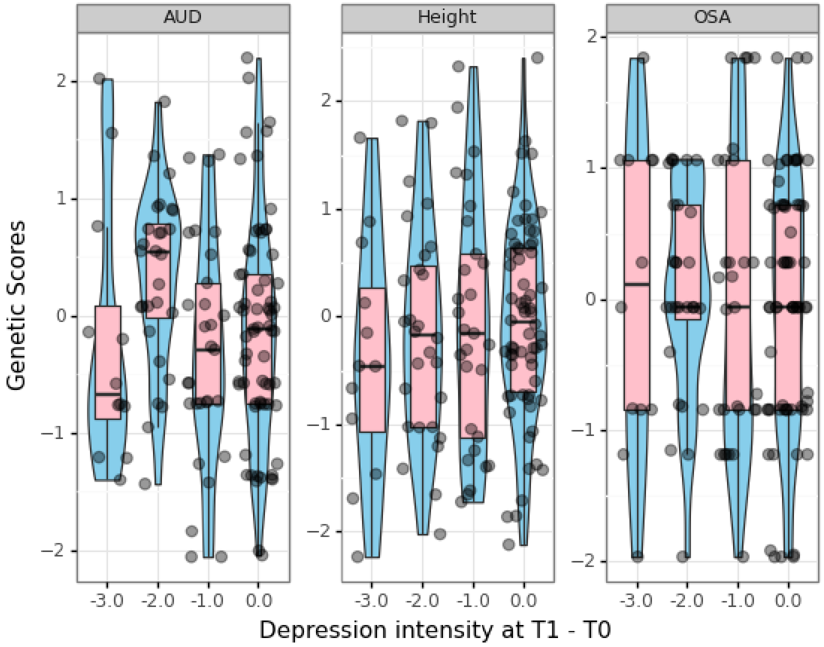

Figure S7. Boxplot of microbiome features associated with improvement on insomnia.

Each panel provides violin plots (sky blue fill), box plots (pink fill), and horizontally jittered points presenting CLR transformed abundances of bacterial genera and functional pathways (y-axis) for each level of improvement of intensity on the outcome measured at T1 - T0 (x-axis). Panel titles display the name of the respective microbiome feature.

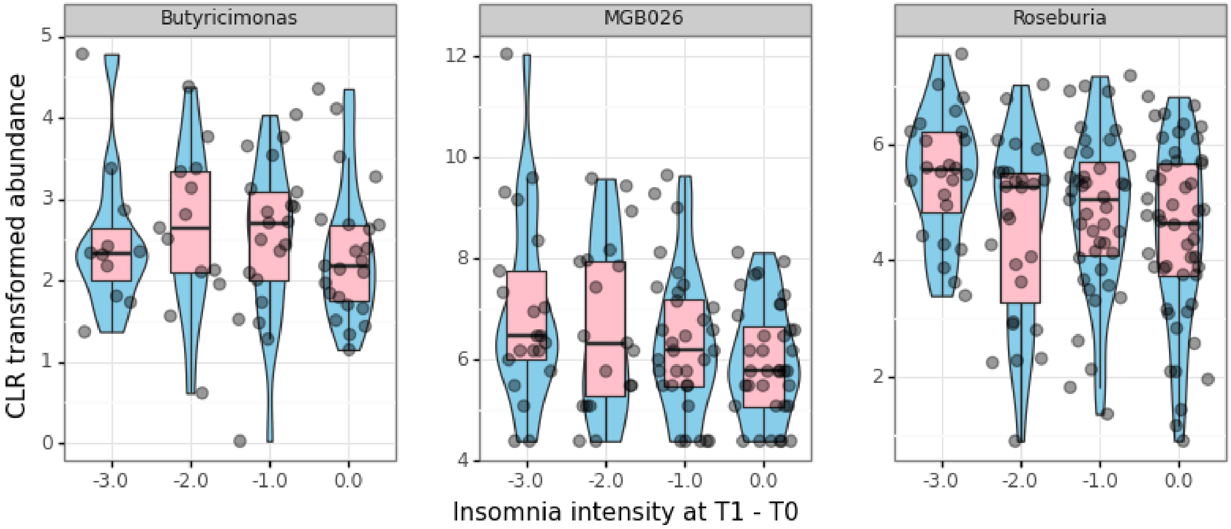

Figure S8. Boxplot of genetic scores associated with improvement on insomnia.

Each panel provides violin plots (sky blue fill), box plots (pink fill), and horizontally jittered points presenting genetic score values (y-axis) for each level of improvement of intensity on the outcome measured at T1 - T0 (x-axis). Panel titles display the name of the respective genetic score.

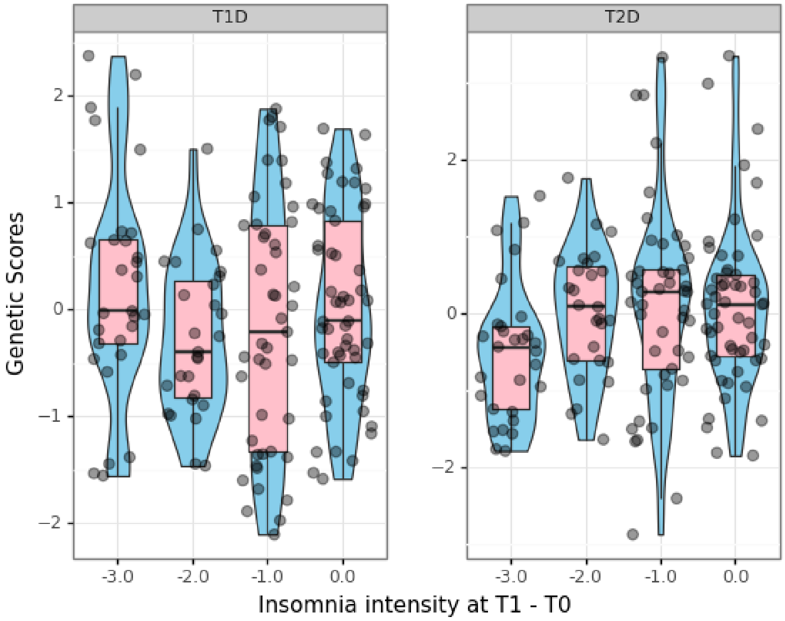

Figure S9. Community-level comparison of the gut microbiome of 328 individuals with and without reported anxiety or depression at baseline.

The figure illustrates a PCoA plot using Bray-Curtis dissimilarity at the genus level. P-values from: PERMANOVA (with strata=age) and beta dispersion test (homogeneity of variances). Ellipses around each group represent a multivariate t-distribution and are based on the variance observed among each group of samples.

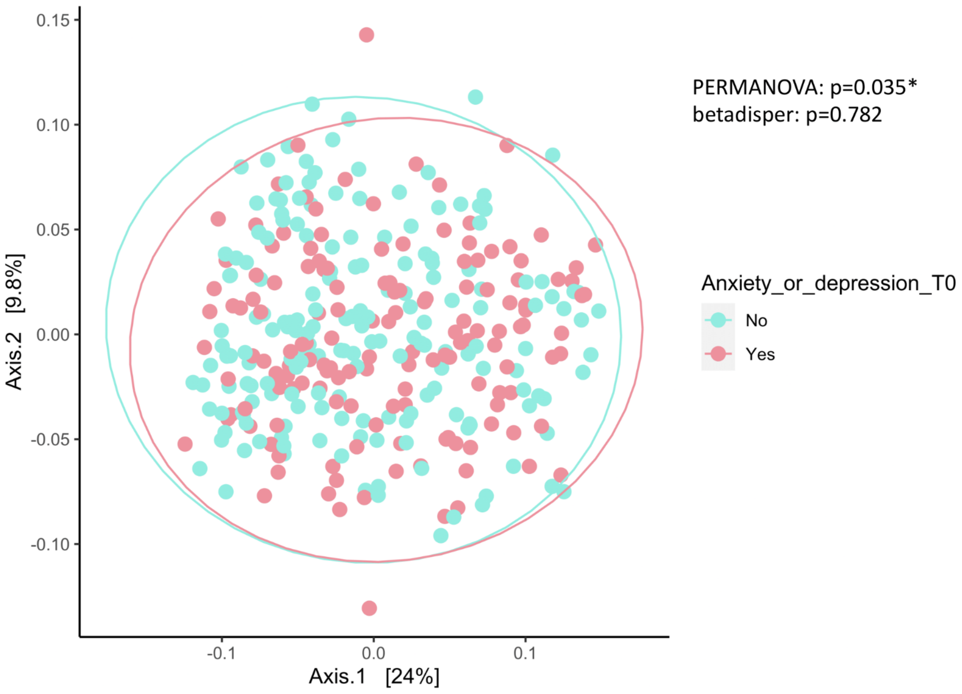

Figure S10. Boxplot of genetic scores associated with anxiety or depression at baseline.

Each panel provides violin plots (sky blue fill), box plots (pink fill), and horizontally jittered points presenting genetic score values (y-axis) for each level of the outcome measuring the reported anxiety or depression at baseline: yes = 1 and no = 0 (x-axis). Panel titles display the name of the respective genetic score.

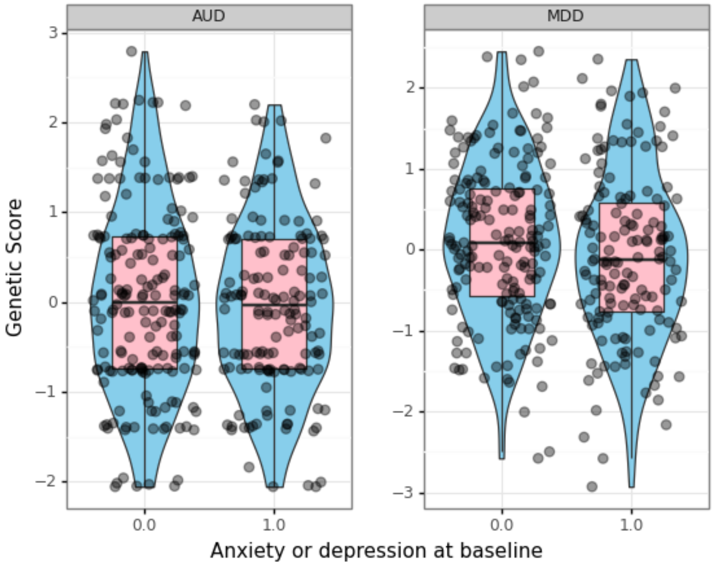

Figure S11. Boxplot of microbiome features associated with sleep problems at baseline.

Each panel provides violin plots (sky blue fill), box plots (pink fill), and horizontally jittered points presenting CLR transformed abundances of bacterial genera and functional pathways (y-axis) for each level of the outcome measuring the reported sleep problems at baseline: yes = 1 and no = 0 (x-axis). Panel titles display the name of the respective microbiome feature.

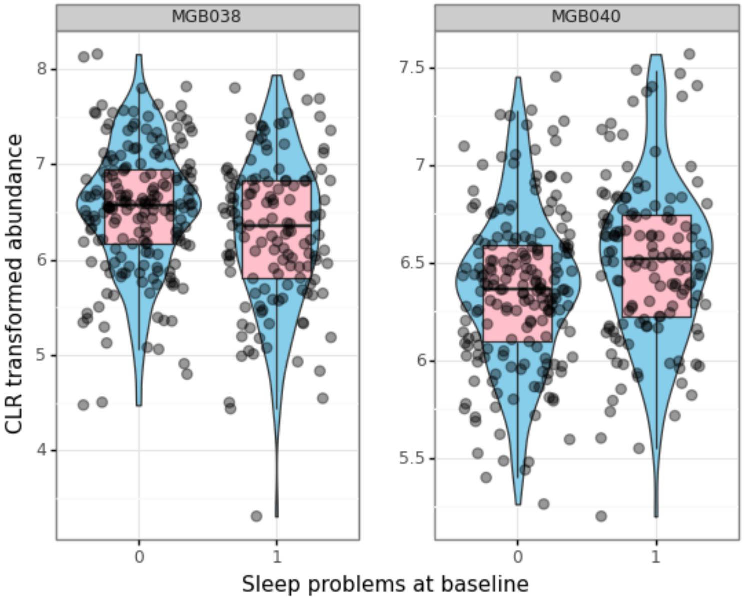

Figure S12 Comparison of models for all outcomes of the study based on demographic, microbiome and genetic factors.

Pseudo r-square values corrected using Pratt's method (y-axis) are shown for each model (x-axis) and outcome (different panels). The vertical line corresponds to the mean ± standard deviation obtained from 1001 bootstrap replicates. D = demographic factors only; D+M = demographic and microbiome factors; D+G = demographic and genetic factors; and D+M+G = all three sets of factors.

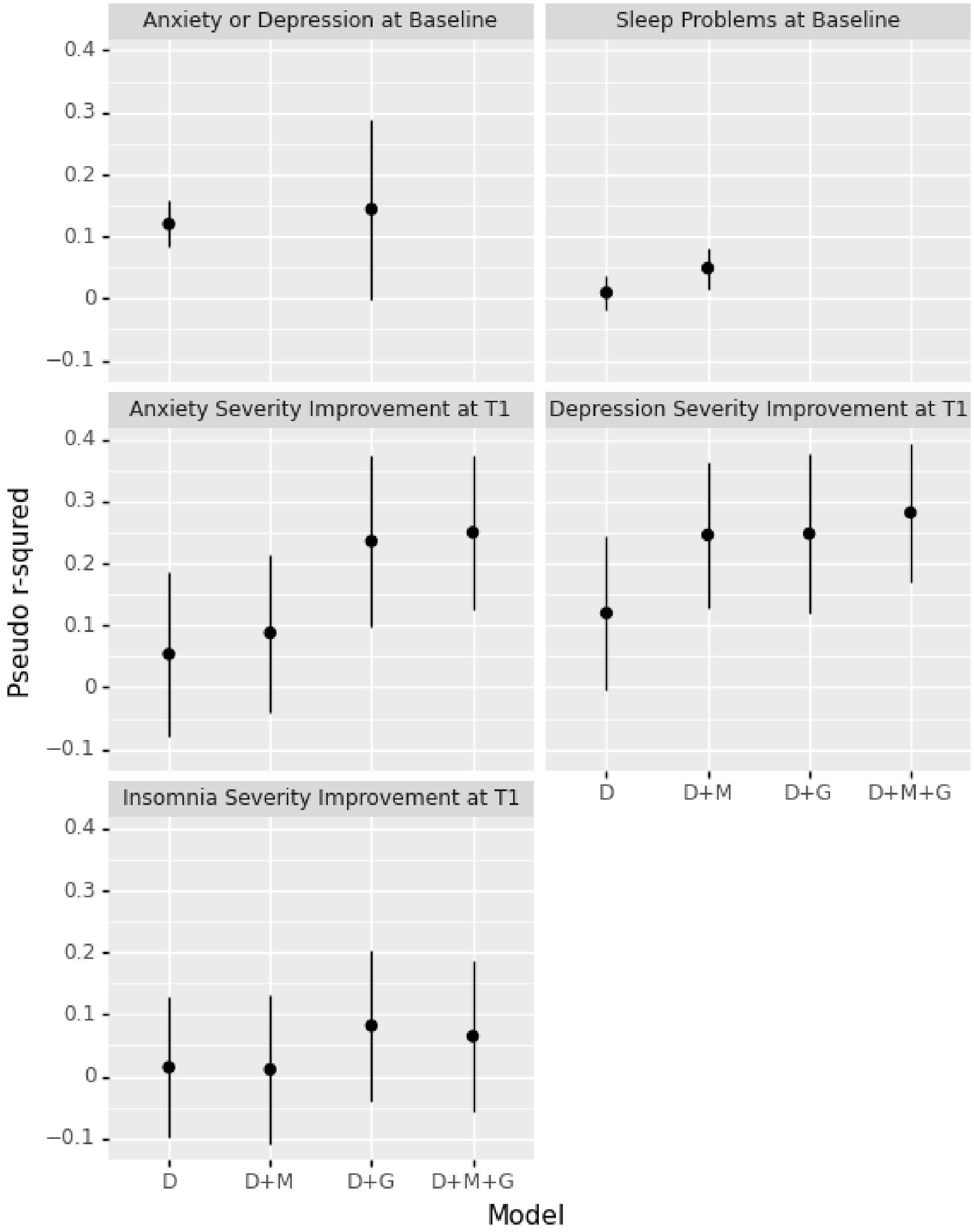

Figure S13. Comparison of functional pathway associations with outcomes at baseline versus improvement at follow-up.

X and Y-axis provide regression coefficients from the models fitted for anxiety or depression, or sleep problems at baseline versus improvement in anxiety (panel A), depression (panel B) or insomnia (panel C) at follow-up with the corresponding standard errors. Dashed line is the line fitted using orthogonal distance regression that accounts for the standard error on x and y variables. If there was a perfect correlation between the variables, the points would lie on the diagonal and the r-squared would be 1. We used a linear model of type y = x*b + a and each panel presents regression coefficients and r-squared values.

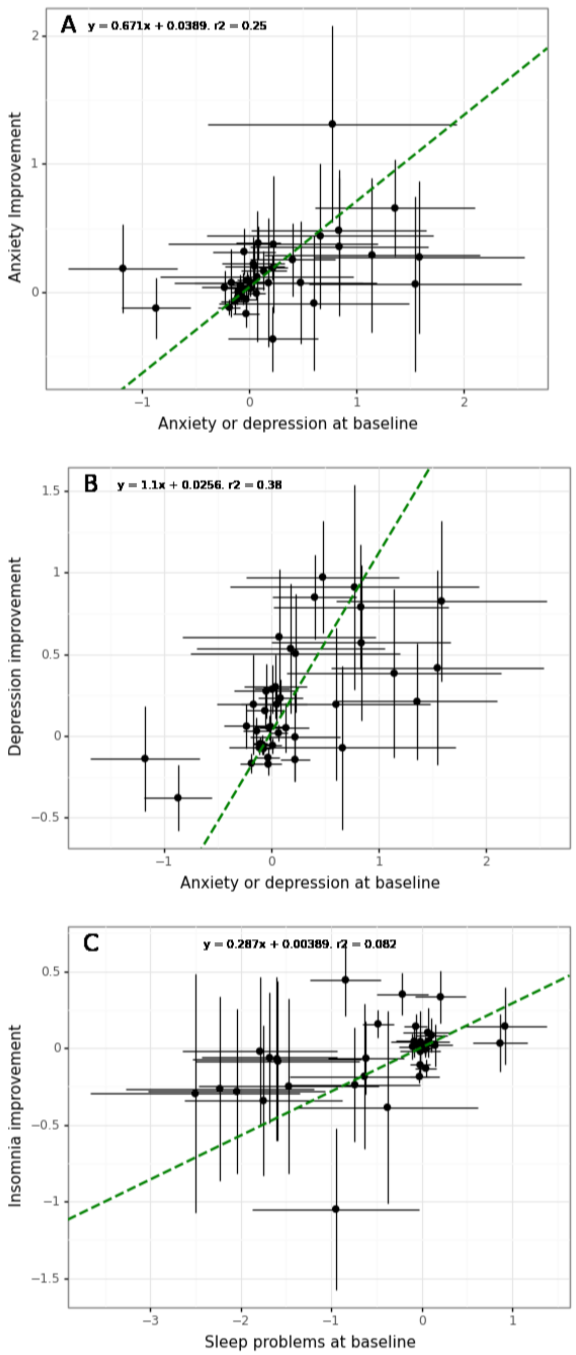
A
y = 0.671x + 0.0389. r2 = 0.25
Anxiety Improvement
Anxiety or depression at baseline
B
y = 1.1x + 0.0256. r2 = 0.38
Depression improvement
Anxiety or depression at baseline
C
y = 0.287x + 0.00389. r2 = 0.082
Insomnia improvement
Sleep problems at baseline

Figure S14. Comparison of bacterial genera associations with outcomes at baseline versus improvement at follow-up.

X and Y-axis provide regression coefficients from the models fitted for anxiety or depression, or sleep problems at baseline versus improvement in anxiety (panel A), depression (panel B) or insomnia (panel C) at follow-up with the corresponding standard errors. Dashed line is the line fitted using orthogonal distance regression that accounts for the standard error on x and y variables. If there was a perfect correlation between the variables, the points would lie on the diagonal and the r-squared would be 1. We used a linear model of type $y = x*b + a$ and each panel presents regression coefficients and r-squared values.

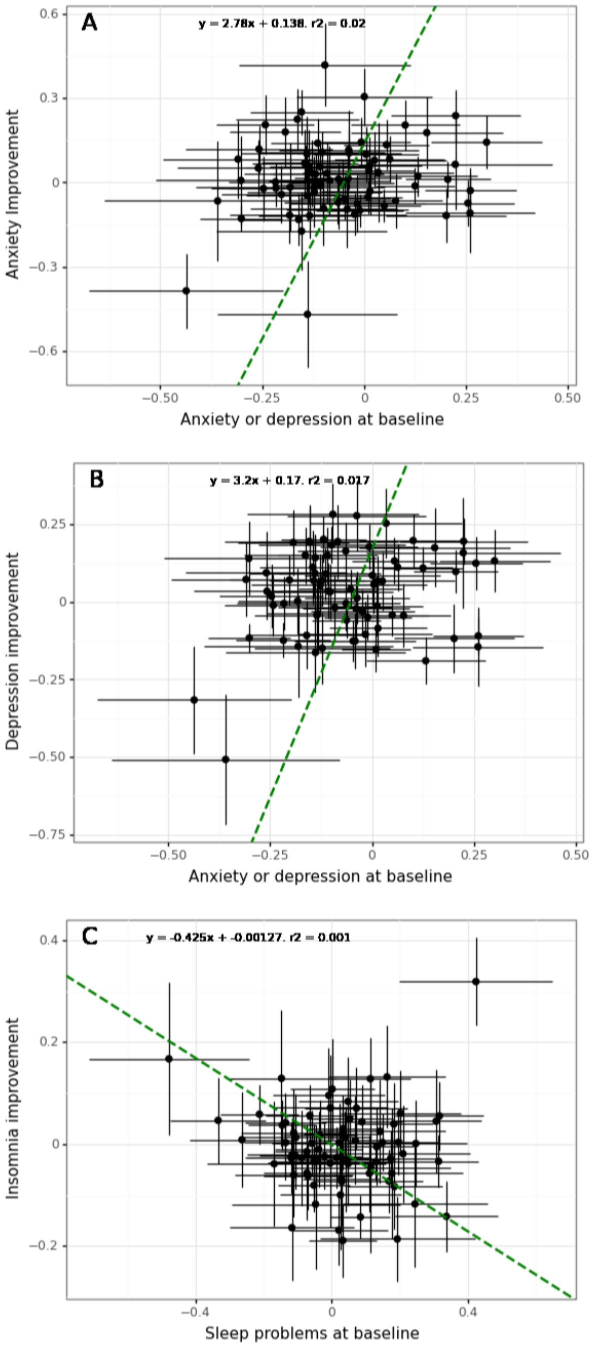
A
y = 2.78x + 0.138. r2 = 0.02
Anxiety Improvement
Anxiety or depression at baseline
B
y = 3.2x + 0.17. r2 = 0.017
Depression improvement
Anxiety or depression at baseline
C
y = -0.425x + -0.00127. r2 = 0.001
Insomnia improvement
Sleep problems at baseline

Figure S15. Comparison of genetic scores associations with outcomes at baseline versus improvement at follow-up.

X and Y-axis provide regression coefficients from the models fitted for anxiety or depression, or sleep problems at baseline versus improvement in anxiety (panel A), depression (panel B) or insomnia (panel C) at follow-up with the corresponding standard errors. Dashed line is the line fitted using orthogonal distance regression that accounts for the standard error on x and y variables. If there was a perfect correlation between the variables, the points would lie on the diagonal and the r-squared would be 1. We used a linear model of type y = x*b + a and each panel presents regression coefficients and r-squared values.

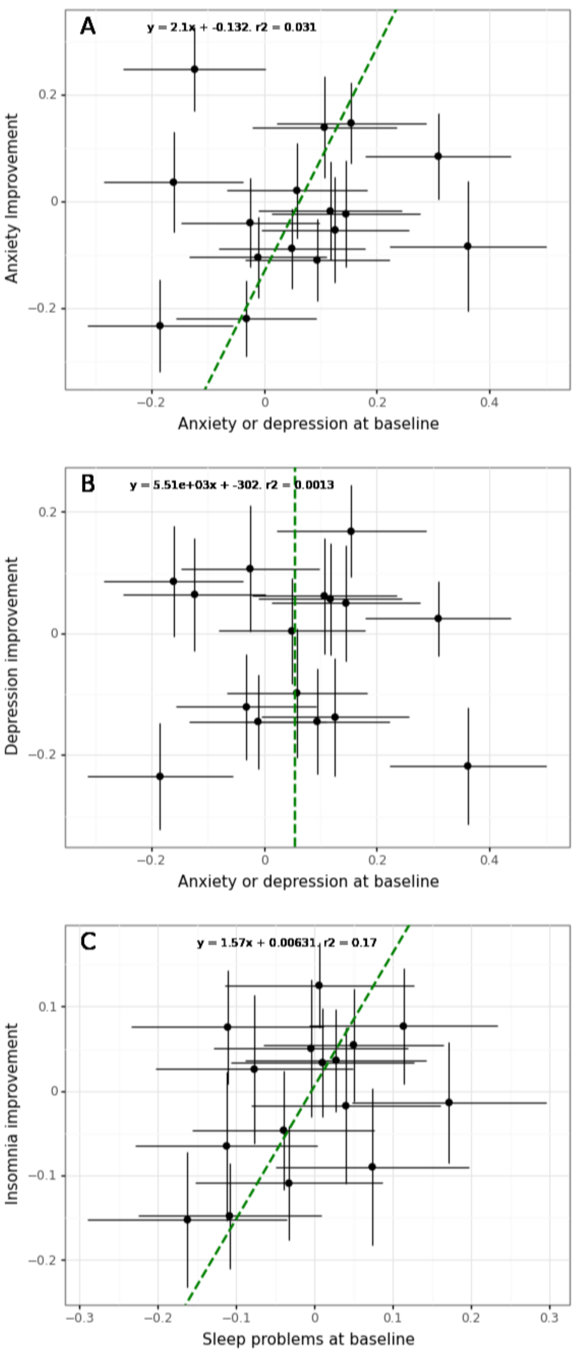
A
y = 2.1x + -0.132. r2 = 0.031
Anxiety Improvement
Anxiety or depression at baseline
B
y = 5.51e+03x + -302. r2 = 0.0013
Depression improvement
Anxiety or depression at baseline
C
y = 1.57x + 0.00631. r2 = 0.17
Insomnia improvement
Sleep problems at baseline

References

1. American College of Gastroenterology. Digestive Health Tips - American College of Gastroenterology. 2021. https://gi.org/topics/digestive-health-tips/. Accessed 6 April 2022.
2. International Foundation for Gastrointestinal Disorders. About GI Motility - About GI Motility. 2021. https://aboutgimotility.org/learn-about-gi-motility/. Accessed 6 April 2022.
3. Davies M, Nowotka M, Papadatos G, Dedman N, Gaulton A, Atkinson F, et al. ChEMBL web services: streamlining access to drug discovery data and utilities. Nucleic Acids Res. 2015;43:W612–W620.
4. Kumbhare SV, Francis-Lyon PA, Kachru D, Uday T, Irudayanathan C, Muthukumar KM, et al. Digital Therapeutics Care Utilizing Genetic and Gut Microbiome Signals for the Management of Functional Gastrointestinal Disorders: Results From a Preliminary Retrospective Study. Front Microbiol. 2022;13:826916.
5. Illumina. Illumina Documentation: 16S Metagenomic Sequencing Library; Preparation, Part # 15044223 Rev. B. 2013.
6. Bolyen E, Rideout JR, Dillon MR, Bokulich NA, Abnet CC, Al-Ghalith GA, et al. Reproducible, interactive, scalable and extensible microbiome data science using QIIME 2. Nat Biotechnol. 2019;37:852–857.
7. Quast C, Pruesse E, Yilmaz P, Gerken J, Schweer T, Yarza P, et al. The SILVA ribosomal RNA gene database project: improved data processing and web-based tools. Nucleic Acids Res. 2012;41:D590–D596.
8. Callahan BJ, Sankaran K, Fukuyama J, McMurdie P, Holmes S. Bioconductor Workflow for Microbiome Data Analysis: from raw reads to community analyses [version 2; peer review: 3 approved]. F1000Research. 2016:1492.
9. Xia Y. q2-repeat-rarefy: QIIME2 plugin for generating the average rarefied table for library size normalization using repeated rarefaction. GitHub; 2021.
10. Aitchison J. The Statistical Analysis of Compositional Data. Stat. Anal. Compos. Data, New York (USA): Chapman and Hall; 1986. p. 416.
11. Douglas GM, Maffei VJ, Zaneveld JR, Yurgel SN, Brown JR, Taylor CM, et al. PICRUSt2 for prediction of metagenome functions. Nat Biotechnol. 2020;38:685–688.
12. Valles-Colomer M, Falony G, Darzi Y, Tigchelaar EF, Wang J, Tito RY, et al. The neuroactive potential of the human gut microbiota in quality of life and depression. Nat Microbiol. 2019;4:623–632.
13. Darzi Y, Falony G, Vieira-Silva S, Raes J. Towards biome-specific analysis of meta-omics data. ISME J. 2016;10:1025–1028.
14. Browning BL, Tian X, Zhou Y, Browning SR. Fast two-stage phasing of large-scale sequence data. Am J Hum Genet. 2021;108:1880–1890.
15. Browning BL, Zhou Y, Browning SR. A One-Penny Imputed Genome from Next-Generation Reference Panels. Am J Hum Genet. 2018;103:338–348.
16. Byrska-Bishop M, Evani US, Zhao X, Basile AO, Abel HJ, Regier AA, et al. High coverage whole genome sequencing of the expanded 1000 Genomes Project cohort including 602 trios. Genomics; 2021.
17. Mölder F, Jablonski KP, Letcher B, Hall MB, Tomkins-Tinch CH, Sochat V, et al. Sustainable data analysis with Snakemake. F1000Research. 2021;10:33.
18. Chang CC, Chow CC, Tellier LC, Vattikuti S, Purcell SM, Lee JJ. Second-generation PLINK: rising to the challenge of larger and richer datasets. GigaScience. 2015;4:7.
19. Pedregosa F, Varoquaux G, Gramfort A, Michel V, Thirion B, Grisel O, et al. Scikit-learn: Machine Learning in Python. J Mach Learn Res. 2011;12:2825–2830.
20. Goodrich J, gnomAD Production Team. Using the gnomAD ancestry principal components analysis loadings and random forest classifier on your dataset. Using GnomAD Ancestry Princ Compon Anal Load Random For Classif Your Dataset. 2021.

https://gnomad.broadinstitute.org/news/2021-09-using-the-gnomad-ancestry-principal-components-analysis-loadings-and-random-forest-classifier-on-your-dataset/. Accessed 15 January 2022.
21. Rajan T, Menon V. Psychiatric disorders and obesity: A review of association studies. J Postgrad Med. 2017;63:182.
22. Simon GE, Von Korff M, Saunders K, Miglioretti DL, Crane PK, van Belle G, et al. Association Between Obesity and Psychiatric Disorders in the US Adult Population. Arch Gen Psychiatry. 2006;63:824.
23. Seabold S, Perktold J. Statsmodels: Econometric and Statistical Modeling with PythonAustin, Texas; 2010. p. 92–96.
24. Zeileis A. Econometric Computing with HC and HAC Covariance Matrix Estimators. J Stat Softw. 2004;11.
25. Benjamini Y, Hochberg Y. Controlling the False Discovery Rate: A Practical and Powerful Approach to Multiple Testing. J R Stat Soc Ser B Methodol. 1995;57:289–300.
26. Claudy JG. Multiple Regression and Validity Estimation in One Sample. Appl Psychol Meas. 1978;2:595–607.
27. Raju NS, Bilgic R, Edwards JE, Fleer PF. Methodology Review: Estimation of Population Validity and Cross-Validity, and the Use of Equal Weights in Prediction. Appl Psychol Meas. 1997;21:291–305.
28. Oksanen J. Vegan: Community Ecology Package. 2020.
